# Immunomodulatory Activities of a Fungal Protein Extracted from *Hericium erinaceus* through Regulating the Gut Microbiota

**DOI:** 10.3389/fimmu.2017.00666

**Published:** 2017-06-12

**Authors:** Chen Diling, Zheng Chaoqun, Yang Jian, Li Jian, Su Jiyan, Xie Yizhen, Lai Guoxiao

**Affiliations:** ^1^State Key Laboratory of Applied Microbiology South China, Guangdong Institute of Microbiology, Guangzhou, China; ^2^Guangdong Provincial Key Laboratory of Microbial Culture Collection and Application, Guangdong Institute of Microbiology, Guangzhou, China; ^3^Guangdong Open Laboratory of Applied Microbiology, Guangdong Institute of Microbiology, Guangzhou, China; ^4^College of Chinese Materia Medica, Guangzhou University of Traditional Chinese Medicine, Guangzhou, China; ^5^College of Chinese Materia Medica, Guangxi University of Traditional Chinese Medicine, Nanning, China; ^6^Guangdong Yuewei Edible Fungi Technology Co., Ltd., Guangzhou, China

**Keywords:** anti-inflammation, functional food ingredient, fungal immunomodulatory protein, gut microbiota, *Hericium erinaceus*, immunotherapy

## Abstract

A single-band protein (HEP3) was isolated from *Hericium erinaceus* using a chemical separation combined with pharmacodynamic evaluation methods. This protein exhibited immunomodulatory activity in lipopolysaccharide-activated RAW 264.7 macrophages by decreasing the overproduction of tumor necrosis factor-α, interleukin (IL)-1β, and IL-6, and downregulating the expression of inducible nitric oxide synthase and nuclear factor-κB p65. Further researches revealed that HEP3 could improve the immune system *via* regulating the composition and metabolism of gut microbiota to activate the proliferation and differentiation of T cells, stimulate the intestinal antigen-presenting cells in high-dose cyclophosphamide-induced immunotoxicity in mice, and play a prebiotic role in the case of excessive antibiotics in inflammatory bowel disease model mice. Aided experiments also showed that HEP3 could be used as an antitumor immune inhibitor in tumor-burdened mice. The results of the present study suggested that fungal protein from *H. erinaceus* could be used as a drug or functional food ingredient for immunotherapy because of its immunomodulatory activities.

## Introduction

Mushrooms are rapidly becoming recognized as a promising source of novel proteins. Fungal immunomodulatory proteins (FIPs) are small-molecule proteins extracted from the fruiting body of some higher basidiomycetes (mushrooms). FIPs have similar structure and immune function as lectins and immunoglobulins, which were first extracted from *Ganoderma lucidum* in 1989. Different kinds of FIPs were extracted from *G. lucidum, G. tsugae* Murrill, *Flammulina velutipes*, and *Volvariella volvacea* continuously (1–4). FIPs have exhibited many beneficial functions in previous studies, including antitumor (5), antiallergy (6, 7), and the ability to stimulate immune cells to produce cytokines (8, 9). Several proteins as lectins (10), lignocellulolytic enzymes (11–14), protease inhibitors (15, 16), and hydrophobins (17–19) have shown unique features and could offer solutions to several medical and biotechnological problems (such as microbial drug resistance, low crop yields, and demands for renewable energy). These stunning properties along with the absence of toxicity render these biopolymers ideal compounds for developing novel functional foods or nutraceuticals with the increase in consumers’ consciousness and demand for healthy food. Large-scale production and industrial application of some fungal proteins prove their biotechnological potential and establish higher fungi as a valuable, although relatively unexplored, source of unique proteins.

*Hericium erinaceus*, belonging to the division Basidiomycota and class Agaricomycetes, is both an edible and medicinal mushroom. It is popular across the continents for its delicacy and is used as a replacement for pork or lamb in Chinese vegetarian cuisine. It is rich in active constituents such as diterpenoid compounds, steroids, polysaccharides, proteins, and other functional ingredients, which are used as good natural plant resources (18). Previous studies have shown the effectiveness of *H. erinaceus* in improving cognitive impairment (20), stimulating nerve growth factors (21) and nerve cells (22), improving hypoglycemia (23), and protecting against gastrointestinal cancers (24, 25). They are also processed into different kinds of products (beverage, cookies, oral liquid, and so on) sold in supermarkets and drugstores. Until now, little has been studied about the proteins from *H. erinaceus* (26). A previous study revealed, using Coomassie Brilliant Blue G-250 method, that the content of total proteins in *H. erinaceus* was up to 20 mg/100 g, indicating that the proteins in *H. erinaceus* might be good active ingredients and hence should not be ignored. Therefore, the aim of this study was to evaluate the immunomodulatory activities of FIPs extracted from the fruiting bodies of *H. erinaceus* using cells and animal experiments and to reveal the underlying mechanism. This study might lay a foundation for the application of the nutritional and medicinal value of *H. erinaceus*.

## Materials and Methods

### Plant Material and Protein Extraction

The fresh fruiting bodies of *H. erinaceus* were collected from the Research Laboratory of Edible Mushrooms of Guangdong Institute of Microbiology, China, in June 2015, and identified by Prof. Xie Yizhen of the Guangdong Institute of Microbiology.

**Figure 1:**
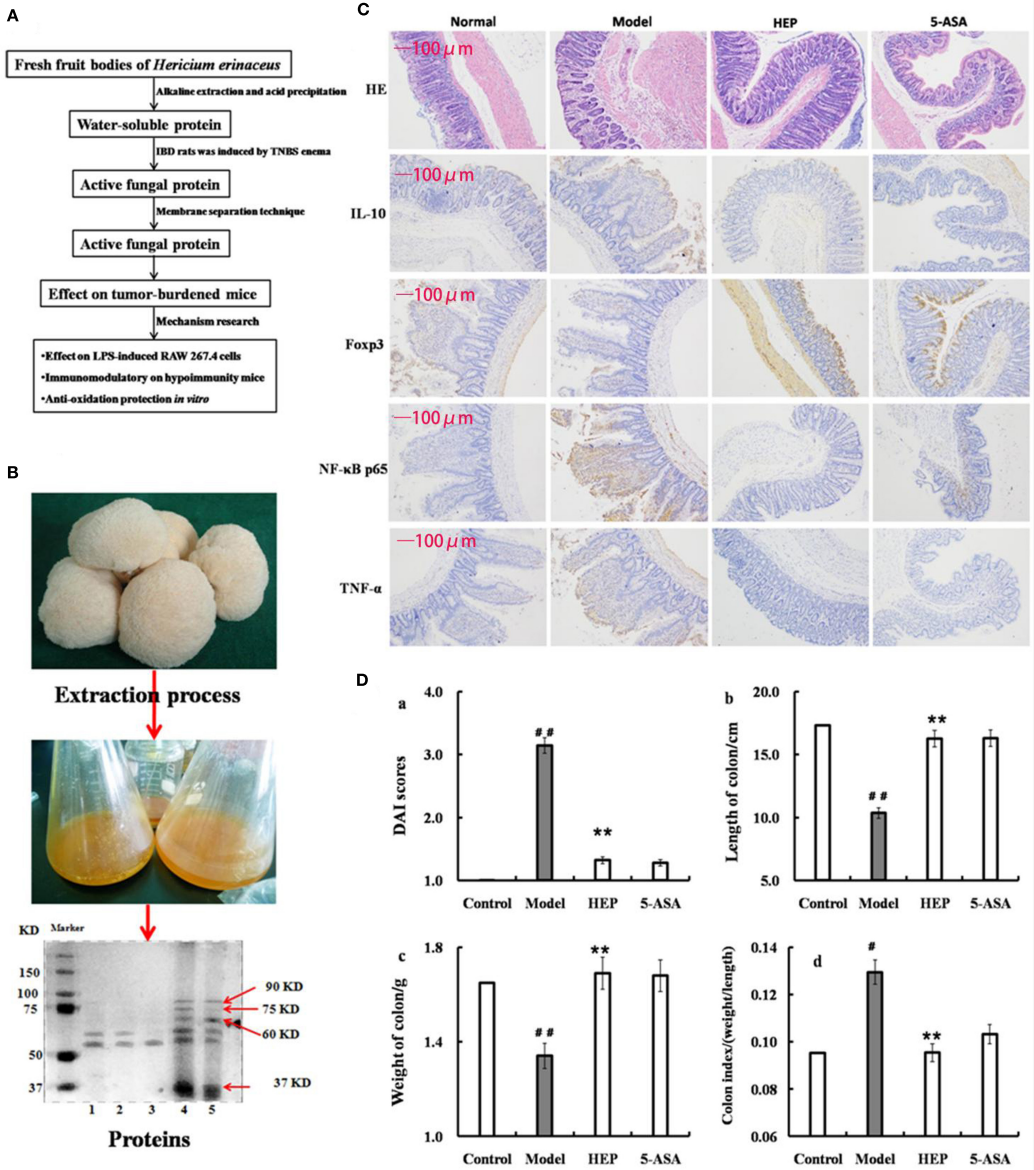
Effect of crude protein extracts from *Hericium erinaceus* on trinitrobenzenesulfonic acid solution (TNBS)-induced inflammatory bowel disease (IBD) rats. **(A)** The technical route of this study; **(B)** the fresh fruiting bodies of *H. erinaceus* and the protein electrophoresis; **(C)** the hematoxylin and eosin-staining and immunohistochemistry results; **(D)** the Disease Activity Index scores (calculated according to the weight loss, stool consistency, and blood in feces) and observation of colons of the TNBS-induced IBD rats. Control is the normal group without any treatments, Model is the TNBS-induced IBD rats, HEP is the crude protein extract-treated group after TNBS enema, and 5-aminosalicylic acid (5-ASA) is the positive control group treated with 100 mg/(kg ⋅ day) of 5-ASA after TNBS enema. Values were expressed as means ± SDs. ^#^*P* < 0.05 vs the control group, *P < 0.05, **P < 0.01 vs the model group, indicating significant differences compared with the model group.

Fresh fruiting bodies (5,000 g) of *H. erinaceus* were pureed in a blender (Philips, HR2095/30, ROYAL PHILIPS, Amsterdam of Holland), and extracts were prepared by the methods shown in the Presentation S1 in Supplemental Material. The solutions were combined, filtrated after acidification to pH 4.3 with dilute acetic acid, and then mixed with (NH_4_)_2_SO_4_ to 80% saturation. The resulting solution was kept in a refrigerator at 4°C overnight and then centrifuged at 5,000 rpm for 20 min at 4°C. The supernatant was removed. The precipitation was dissolved in 5 mL of pH 8.0 Tris–HCl buffer and lyophilized in a vacuum freeze dryer (Alphai-4LD plus, Marin Christ, Osterode, Germany) for crude protein extraction (Figure 1A). The next purification was done using the membrane separation technology combined with the activity evaluation experiment in rats with trinitrobenzenesulfonic acid solution (TNBS)-induced inflammatory bowel disease (IBD).

The protein extracts were analyzed by sodium dodecyl sulfate-polyacrylamide gel electrophoresis (SDS-PAGE). SDS-PAGE (10% w/v) was performed on a Mini-PROTEAN II gel apparatus (Bio-Rad Laboratories, Inc., USA) as described by Laemmli and Favre (27). The gels were stained with Coomassie Brilliant Blue R-250, and protein molecular weight standard (Amersham Biosciences, Sweden) was used as a reference. As shown in Figure 1B, the extracts contained many kinds of proteins, with the majority having a molecular weight of 37–100 kDa; some had a molecular weight of 50–60 kDa (HEP3, Figure 1B). The proteins were isolated and purified using the membrane separation technology combined with Sephadex G-75 chromatography (Sigma-Aldrich Co. LLC, USA).

### Animals

This study used 5- to 6-week male Sprague-Dawley rats (weighing 180–220 g), 4- to 5-week male BALB/c mice (weighing 16–20 g), and Kunming male mice (weighing 18–22 g); all purchased from the Animal Center of the Guangdong Medical Laboratory Animal Center, Guangzhou, China. The animals were kept in the specific-pathogen-free Animal Laboratory of Guangdong Institute of Microbiology, in a temperature (23 ± 1°C) and humidity (55 ± 10%) controlled room under a 12-h light/dark cycle (lights off at 1700 p.m.). The animals were given free access to food and water that were sterilized. The experimental protocols were approved by the Animal Ethics Committee of Guangdong Institute of Microbiology, and all experimental procedures conformed to the National Institutes of Health Guide for the Care and Use of Laboratory Animals. All efforts were made to minimize the number of animals used.

### Cell Culture

The RAW 264.7 macrophages, HIEpiC, and CC531 cell lines were obtained from the Shanghai Aolu Biological Technology Co., Ltd. (China). They were maintained in Dulbecco’s modified Eagle medium or RPMI-1640 supplemented with 10% fetal bovine serum at 37°C in a humidified atmosphere of 95% air and 5% CO_2_ and seeded into a 75-cm^2^ culture dish. On reaching 80% confluence, the cells underwent digestive transfer culture after fusion growth at a density of 5 × 10^4^ cells/mL.

### Anti-inflammatory Evaluation of IBD Model Rats

After 7 days of adaptation period, the animals were randomly divided into four groups [100 mg/(kg ⋅ day): proteins extracted from *H. erinaceus* (HEP), model, normal, and 5–aminosalicylic acid groups], with six rats in each group, and housed three per cage. The rats were fed a standard diet, and water was available freely. After 24 h of fasting, the rats were anesthetized by intraperitoneally injecting 2% sodium pentobarbital (0.2 mL/100 g). The rats were intubated (using latex tubing of 2 mm diameter, lubricated with edible oil before use) from the anus, gently inserting the tubing into the lumen about 8.0 cm. Then, 150 mg/kg of TNBS (dissolved in 50% ethanol; Sigma-Aldrich, MO, USA) solution was injected through the latex tubing, and the rats were hung upside down for 30 s to enable the mixture to fully seep into the lumen without leakage. The rats in the HEP group were treated by intragastric administration after 1 day of TNBS induction.

After 14 days of treatment, the rats were anesthetized by intraperitoneally injecting 2% sodium pentobarbital (0.25 mL/100 g). The blood plasma was collected by the abdominal aortic method, and the serum by centrifugation (1,500 rpm, 10 min). Then, the serum was used to monitor the production of the cytokines interleukin (1L)-1α, 1L-2, 1L-8, 1L-10, 1L-11, and IL-12; tumor necrosis factor (TNF)-γ and TNF-α; vascular endothelial growth factor (VEGF); human macrophage inflammatory protein-1α (MIP-α); and macrophage colony-stimulating factor (M-CSF) and myeloperoxidase (MPO). The colons obtained from the rats were fixed in 4% paraformaldehyde at pH 7.4 for further pathological observation.

### Immunomodulatory Activity on RAW 264.7 Macrophages

After incubating RAW 264.7 macrophages with HEP3 (0–200 μg/mL) for 4 h, followed by an additional 24 h of treatment with lipopolysaccharide (LPS; 1 μg/mL), the supernatant was used to monitor the production of the cytokines 1L-1β, 1L-6, TNF-α, and nitric oxide (NO), and the intracellular levels of inducible nitric oxide synthase (iNOS) and nuclear factor-κB (NF-κB) p65. The assays were carried out according to the procedures recommended in the enzyme-linked immunosorbent assay (ELISA) kit manual, which was purchased from USCN Life Science Inc. (Wuhan, China).

### Effect on the Cyclophosphamide Immunosuppressant Mice Model

The animals were randomly divided into four groups (*n* = 10): normal, model, and HEP3-treated with 200 and 100 mg/(kg ⋅ day) groups. The immunosuppressant mice were induced by intraperitoneally injecting cyclophosphamide [cyclophosphamide-induced group (CTX), 80 mg/kg] once a day, for 3 days, while the mice in the normal group were intraperitoneally injected with saline as a control. All mice had free access to tap water and food (*ad libitum*). On day 14, the mice were sacrificed, and the serum, spleen, and cecal contents were isolated for further analysis.

### Prebiotic Effect of HEP3 on TNBS-Induced Mice

All animals were randomly divided into nine groups (*n* = 9): control, model, model and high-dose antibiotics, HEP3 [100 mg/(kg ⋅ day)], *Bifidobacterium*, HEP3 and high-dose antibiotics, HEP3 and *Bifidobacterium, Bifidobacterium* and high-dose antibiotics, and HEP3 and *Bifidobacterium* and high-dose antibiotics. All the antibiotics were given for 4 days. Then, IBD was induced with TNBS, followed by 7 days of drug treatment and induction with TNBS again, and finally followed by another 4 days of drug treatment. The model mice were prepared using TNBS (150 mg/kg) enema according to the procedure described in Section “Anti-inflammatory Evaluation of IBD Model Rats.”

After treatment, the mice were anesthetized by intraperitoneally injecting 2% sodium pentobarbital (0.25 mL/100 g). The blood plasma was collected by the abdominal aortic method, and the serum by centrifugation (1,500 rpm, 10 min). Then, the serum was used to monitor the production of cytokines granulocyte-macrophage colony-stimulating factor (GM-CSF), TNF-γ, 1L-10, IL-12, 1L-17α, 1L-4, TNF-α, and VEGF. The colons and spleens obtained from the rats were fixed in 4% paraformaldehyde at pH 7.4 for further pathological observation, and the cecum contents were collected for 16s rRNA analysis.

### Antiaging Protective Effect on the d-Galactose-Induced Senescent Cells

The HIEpiC cells were induced by 40 g/L d-galactose for 72 h and co-incubated with or without different concentrations of HEP (0–200 μg/mL). The methyl thiazolyl tetrazolium (MTT) assay was conducted to assess the cell viability. Senescence-associated β-galactosidase staining (operational procedure according to the kits’ instructions) was used to identify the senescent cells. The activities of malondialdehyde (MDA), total superoxide dismutase (T-SOD), and glutathione peroxidase (GSH-Px) were measured. The protein concentration of cells was determined using the Coomassie Brilliant Blue G250 assay. The enzyme activities, level of MDA, and protein content were all determined using the detection kits purchased from the Nanjing Jiancheng Bioengineering Institute (Nanjing, Jiangsu, China). The procedures were performed according to the manufacturer’s instruction. The levels were normalized to the protein concentration of each sample and expressed as a percentage of non-treated controls.

### Antitumor Experiment

The CC531 cells were cultured in the RPMI-1640 medium (containing 10% calf serum), placed in an incubator at 37°C with 5% CO_2_ and saturated humidity. The culture medium was replaced every 2 days, and the adherent cells were digested using 0.05% trypsin when the cells reached 80% confluence after 7 days of adaptation period. The logarithmic-phase human prostate cancer cell line CC531 was prepared to a concentration of 1.0 × 10^7^ cells/mL. Each mouse was injected subcutaneously with 0.2 mL of cell suspension.

Two weeks later, the minimum and maximum diameters of the tumor body were measured. Then, 24 moderately sized mice were chosen and divided into three groups, including HEP3 high-dose group [HH, 100 mg/(kg ⋅ day)], HEP3 low-dose group [HL, 50 mg/(kg ⋅ day)], and model group, with eight mice in each group, and another eight normal mice as the normal group. The volume of the dose was 0.2 mL per mice per day. The model and normal groups were given equivalent volume of phosphate-buffered saline (PBS). Three weeks later, the rats were anesthetized by intraperitoneally injecting 2% sodium pentobarbital (0.25 mL/100 g), decapitated, and dissected. The blood plasma was collected from the orbit, and the serum by centrifugation (1,500 rpm, 10 min). Then, the serum was used to monitor the production of tumor-associated cytokines TNF-α, interferon (IFN)-γ, M-CSF, transforming growth factor (TGF), and VEGF. All the assays were carried out according to the procedures recommended in the ELISA kit manual. The mice were sacrificed by cervical dislocation. The tumor tissue was stripped off, and the tumor inhibition rate (TIR) was calculated. The sample was stored in liquid nitrogen for further use.

### Microbiome Analysis

Fresh fecal samples were collected before the fasting of the rats and stored at −80°C. Frozen microbial DNA isolated from mice cecal sample with the total mass ranging from 1.2 to 20.0 ng was stored at −20°C. The microbial 16S rRNA genes were amplified using the forward primer 5′-ACTCCTACGGGAGGCAGCA-3′ and the reverse primer 5′-GGACTACHVGGGTWTCTAAT-3′. Each amplified product was concentrated *via* solid-phase reversible immobilization and quantified by electrophoresis using an Agilent 2100 Bioanalyzer (Agilent, USA). After quantifying DNA concentration using NanoDrop spectrophotometer, each sample was diluted to a concentration of 1 × 10^9^ mol/μL in the Tris–EDTA buffer and pooled. Then, 20 μL of the pooled mixture was used for sequencing with the Illumina MiSeq sequencing system according to the manufacturer’s instructions. The resulting reads were analyzed as described in a previous study (28).

### Hematoxylin and Eosin (HE) Staining and Immunohistochemical Analysis

Tissues from the mice or rats were freshly excised and fixed in 10% triformol. Once the samples were fixed, dehydration, clarification, and inclusion were carried out. After the blocks were obtained, the sections were cut using a microtome (Microm HM325, Germany), with a thickness of 5 μm. Sections of hydrated and deparaffinized tissues were stained with HE followed by appropriate method for histological observation. From each colon description, 10 sections were analyzed by three independent observers (JM, EM, and RMC).

The paraffin-embedded slices of colon tissue (4 μm) were incubated overnight with anti-NF-κB p65, anti-Foxp3, anti-IL-10, and anti-TNF-α primary antibodies at 4°C; all the antibodies were purchased from Abcam (Cambridge, UK). The slices were then washed with PBS and incubated with horseradish peroxidase-conjugated secondary antibody for 1 h at room temperature. After washing with PBS again, the slices were developed using 3,3′-diaminobenzidine as a chromogen and counterstained with hematoxylin. Images were acquired using a Leica DM2500 system (Leica Microsystems, Germany).

### Statistical Analysis

All data were expressed as means plus SDs of at least three independent experiments. The significant differences between treatments were assessed with one-way analysis of variance or Student’s *t-*test at *P* < 0.05 using the Statistical Package for the Social Sciences (SPSS; Abacus Concepts, CA, USA) and Prism 5 (GraphPad, CA, USA) software.

## Results

### Anti-inflammatory Effect on IBD Model Rats

**Figure 2:**
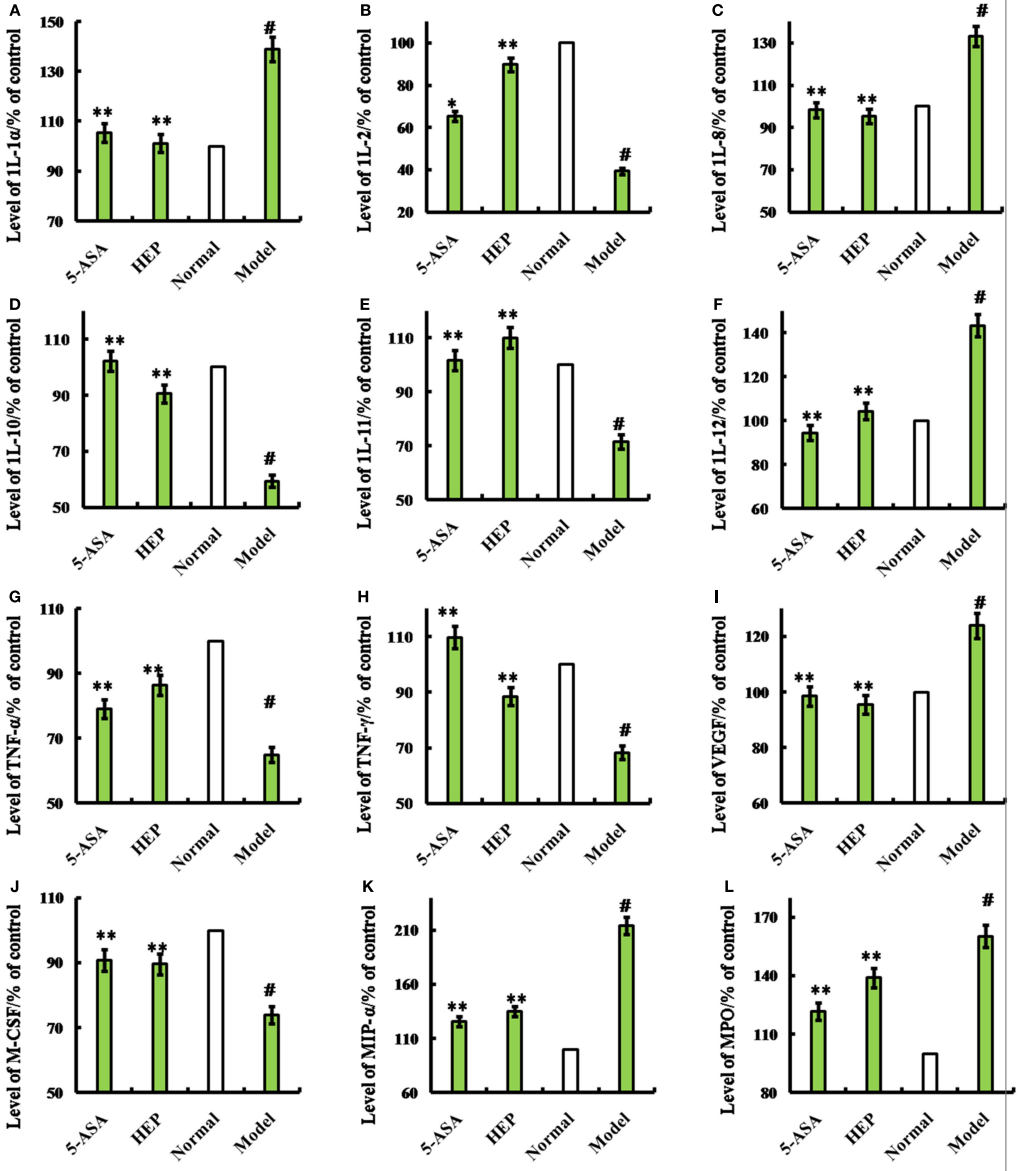
Effects of HEP on trinitrobenzenesulfonic acid solution (TNBS)-induced rats. Normal group; model group, induced by TNBS enema; HEP group, the crude protein extract-treated group after TNBS enema; and positive control group, treated with 100 mg/(kg ⋅ day) of 5-aminosalicylic acid after TNBS enema. After treatment for 14 days, cytokines interleukin (1L)-1α **(A)**, 1L-2 **(B)**, 1L-8 **(C)**, 1L-10 **(D)**, 1L-11 **(E)**, IL-12 **(F)**, tumor necrosis factor (TNF)-γ **(H)**, TNF-α **(G)**, vascular endothelial growth factor (VEGF) **(I)**, MIP-α **(J)**, macrophage colony-stimulating factor (M-CSF) **(K)**, and myeloperoxidase (MPO) **(L)** were produced. The assays were carried out according to the procedures recommended in the enzyme-linked immunosorbent assay kit manual. Values were means ± SDs of three independent experiments. ^#^*P* < 0.05 vs the normal group, *P < 0.05, **P < 0.01 vs the TNBS-treated group.

An IBD rat model was prepared to evaluate the immune enhancement effect of HEP (crude protein from *H. erinaceus*). After treatment with TNBS enema, the rats in all groups except the control displayed anepithymia with reduced activity, lethargy, and ruffled fur, along with bloody stools or stools containing occult blood, and weight loss. However, these symptoms disappeared from day 9 or 10. The results of the experimental treatments in terms of the Disease Activity Index are shown in Figure 1D. The rats in the HEP-treated group showed a significant improvement compared with the TNBS-treated group. Massive inflammatory cell infiltration was observed in the colonic mucosa and submucosa of TNBS-induced rats under a light microscope. Treatment of HEP did not relieve this inflammatory phenomenon, but it reduced the number of inflammatory cells obviously (Figure 1C). All sections were observed under the same conditions using light microscopy (Figure 1C). Brown particles were considered as positive cells. The percentage of Foxp3- and IL-10-positive cells in rats in the model group was significantly lower than the normal (*P* < 0.05), while the percentage of TNF-α and NF-κB p65 was significantly higher (*P* < 0.05). After treatment with HEP, the percentages of Foxp3- and IL-10-positive cells significantly increased compared with the model group, and the percentages of TNF-α- and NF-κB p65-positive cells significantly reduced compared with the model group (*P* < 0.05). After treatment with 100 mg/(kg ⋅ day) of HEP, all the cytokine levels were restored to near normal; some anti-inflammatory cytokines 1L-1α (Figure 2A), 1L-2 (Figure 2B), 1L-8 (Figure 2C), 1L-10 (Figure 2D), 1L-11 (Figure 2E), IL-12 (Figure 2F), TNF-γ (Figure 2H), TNF-α (Figure 2G), VEGF (Figure 2I), MIP-α (Figure 2K), M-CSF (Figure 2J), and MPO activity (Figure 2L) were secreted significantly and better compared with the positive control group (*P* < 0.05), as shown in Figure 2. Cumulatively, all these results suggested that HEP had an effective anti-inflammatory effect on IBD mice.

### HEP3 Is a FIP in LPS-Activated RAW 264.7 Macrophages

**Figure 3:**
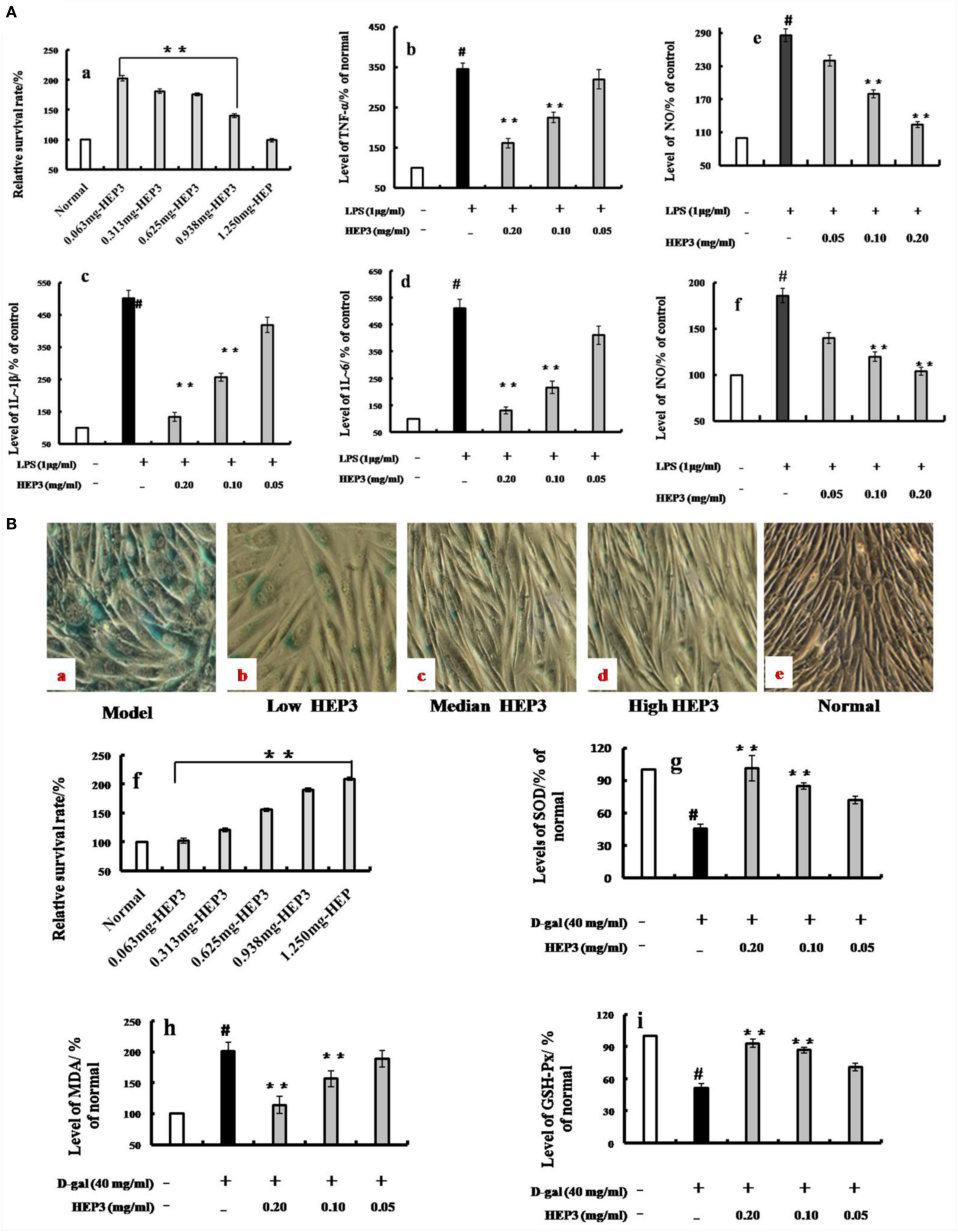
Effects of HEP3 on proinflammatory cytokine productions in lipopolysaccharide (LPS)-activated RAW 264.7 cells, and effects on the d-galactose-induced HIEpiC senescent cells. Cell viability was measured by quantitative colorimetric methylthiazolyl tetrazolium (MTT) after incubation with HEP3 for 48 h [**(A)**, a]; cells were preincubated with 0.05–0.20 mg/mL HEP3 for 4 h, and then treated with 1 μg/mL LPS for 24 h. Using an ELISA kit, tumor necrosis factor-α [**(A)**, b], interleukin (IL)-1β [**(A)**, c], IL-6 [**(A)**, d], nitric oxide [**(A)**, e], and inducible nitric oxide synthase [**(A)**, f] in the supernatant were detected. [**(B)**, a–e] The cells were treated with 40 mg/mL of d-galactose for 72 h combined with different concentrations of HEP3, and the number of senescent cells (blue-stained cells) was detected using β-galactosidase staining. [**(B)**, f] The cells were treated with different concentrations of HEP for 24 h, and the cytotoxicity was detected by an MTT assay. [**(B)**, g–i] The activities of malondialdehyde, total superoxide dismutase, and glutathione peroxidase of the cells. Values were means ± SDs of three independent experiments. ^#^*P* < 0.05 vs the normal group, *P < 0.05, **P < 0.01 vs the model group, indicating significant differences compared with the model group.

A membrane separation technology method was used, and a single-band protein (HEP3, Figures 1B, 2 and 3) was isolated and purified to further target the active protein in *H. erinaceus*. Then, the RAW 264.7 macrophages were used to further evaluate the immunomodulatory activities. The results showed that after incubating the RAW 264.7 macrophages with HEP3 for 12 h and an additional 12 h of treatment with LPS (1 μg/mL), TNF-α production was significantly stimulated and 1L-1β and 1L-6 were also found to be significantly induced, as shown in Figure 3A. However, the overproduction of TNF-α (Figure 3A, b), IL-1β (Figure 3A, c), and IL-6 (Figure 3A, d) considerably reduced by 0.05–0.20 mg/mL HEP3 treatment, indicating that HEP3 was able to suppress the LPS-induced production of inflammatory cytokines in the RAW 264.7 macrophages. The HEP3 did not show any harmful effect at a concentration of 1.25 mg/mL. The results also revealed that HEP3 at 0.05–0.20 mg/mL perfectly suppressed NO secretion (Figure 3A, e), with no significant difference compared with the control at high concentration. HEP3 (0.05–0.20 mg/mL) significantly inhibited the LPS-induced iNOS expression (Figure 3A, f). It is suggested that HEP3 probably suppressed NO secretion by downregulating the expression of iNOS in the LPS-stimulated RAW 264.7 macrophages. All the results revealed that HEP3, a 52-kDa protein extracted from *H. erinaceus*, was a FIP.

### HEP3 Reversed d-Galactose-Induced HIEpiC Senescent Cell Proliferation

As shown in Figure 3B, the number of blue-stained cells of the model group [induced by 40 mg/mL of d-galactose for 72 h (Figure 3B, a); the d-galactose-induced senescent cells are not shown] was obviously higher than that of the normal group (*P* < 0.05); HEP3 could reduce the number of senescent cells, especially in the high-dose group, and promote cell proliferation (Figure 3B, b–e). The antioxidant protection activity was assessed by measuring the intracellular levels of MDA, GSH-Px, and SOD. After exposure of the cells to 40 mg/mL of d-galactose for 72 h, the intracellular MDA level was significantly elevated to 201% of the control value, while GSH-Px and SOD levels were substantially attenuated to 51.2 and 45.6% of the control value, suggesting that d-galactose induced marked oxidative stress. When the cells were co-incubated with HEP3 at concentrations of 0.05, 0.10, and 0.20 mg/mL, the intracellular MDA production significantly reduced (189, 156, and 114% of the control value, respectively; Figure 3B, h) compared with the d-galactose group. However, HEP also increased the GSH-Px (72, 85, and 101% of the control value, respectively; Figure 3B, i) and SOD levels (71, 87, and 93% of the control value, respectively; Figure 3B, g) compared with the d-galactose group. These experimental findings indicated that HEP3 treatment could significantly reduce the d-galactose-induced oxidative stress on the HIEpiC cells.

### HEP3 Ameliorated Cyclophosphamide-Induced Immunotoxicity in Mice

#### Improvement in Clinical Parameters

**Figure 4:**
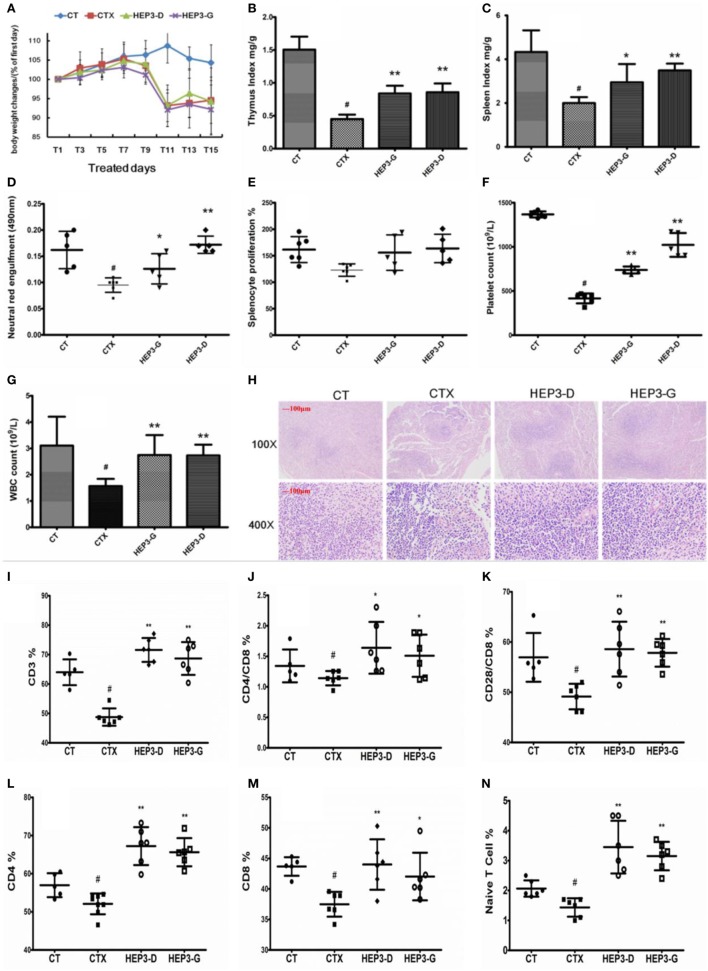
Effect of HEP3 on the cyclophosphamide-induced immunotoxicity mice. Body weight changes **(A)**, thymus index **(B)**, and spleen index **(C)**, neutral red engulfment **(D)**, splenocyte proliferation **(E)**, platelet **(F)**, and white blood cell **(G)**, the tissue structure of the spleen **(H)**, the CD3^+^ **(I)**, CD4^+^/CD8 **(J)**, CD4^+^ **(L)**, CD8^+^ **(M)**, CD28^+^/CD8 **(K)**, and naive T cells **(N)**. CT is the control group treated with just vehicle, CTX is the cyclophosphamide-induced group (intraperitoneal injection of 80 mg/kg) group, HEP3-D is the group treated with 100 mg/kg HEP3 and intraperitoneal injection of 80 mg/kg cyclophosphamide, and HEP3-G is the group treated with 200 mg/kg HEP3 and intraperitoneal injection of 80 mg/kg cyclophosphamide. Values were means ± SDs of six independent experiments. ^#^*P* < 0.05 vs the control group, *P < 0.05, **P < 0.01 vs the CTX, indicating significant differences.

The immune response of mice with high-dose cyclophosphamide-induced immunotoxicity was monitored to further understand the immunomodulatory activity of the protein extracted from *H. erinaceus*. As shown in Figure 4, all the immune indexes, including thymus (Figure 4B) and spleen (Figure 4C) index, platelet (Figure 4F) and white blood cell (Figure 4G), neutral red engulfment (Figure 4D), and splenocyte proliferation (Figure 4E), were enhanced (*P* < 0.05) compared with the CTX; the tissue structure of the spleen also improved (Figure 4H). Moreover, the CD3^+^ (Figure 4I), CD4^+^ (Figure 4L), CD8^+^ (Figure 4M), CD28^+^ (Figure 4K), and naive T cells (Figure 4N) were measured using the flow cytometry (FACS Calibur, Becton Dickinson, USA). All the mentioned parameters were activated compared with the high-dose CTX (*P* < 0.05), indicating that HEP3 could activate the T cells. The results of the present study showed that the HEP3 could reverse the high-dose cyclophosphamide-induced immunotoxicity in mice.

#### Recapitulating the Gut Microbiota Composition

**Figure 5:**
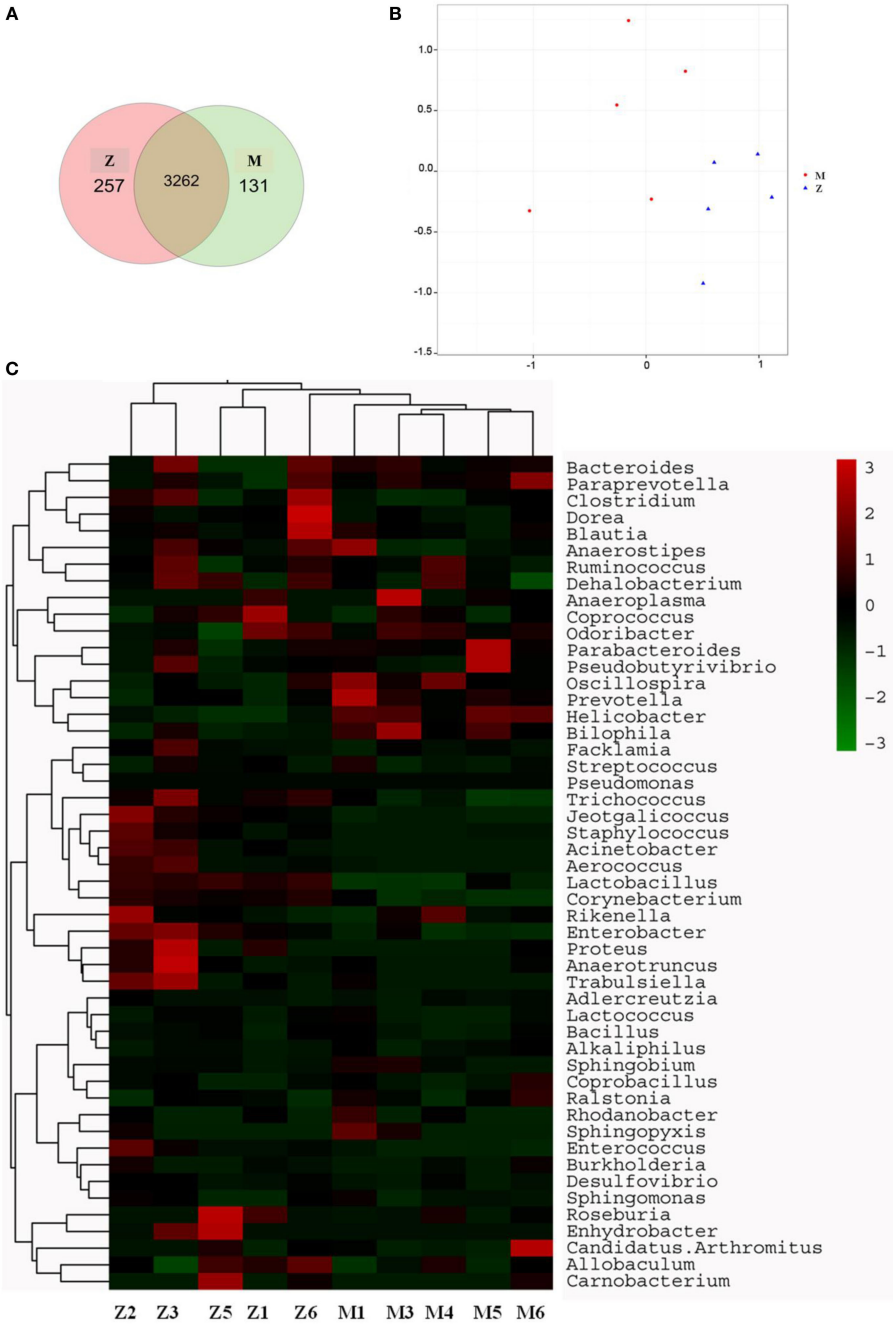
Influence of cyclophosphamide on the cecal microbiota of mice. **(A)** The Venn diagram; **(B)** the PCA analysis of operational taxonomic units; **(C)** heat map of 16S rRNA gene sequencing analysis of cecal content at the genus level. Z denotes the control (normal) group just treated with vehicle, and M is the cyclophosphamide-induced (intraperitoneal injection of 80 mg/kg) group.

The gut microbiota was proved to have a significant influence on the immune system of organisms. The changes in gut microbiota in the high-dose cyclophosphamide-induced group and normal group mice are shown in Figure 5. The Venn (Figure 5A), principal component analysis (PCA; Figure 5B), and heatmap (Figure 5C) results showed that the high-dose cyclophosphamide changed the gut microbiota composition obviously compared with the normal group, as the relative abundances at the genus level of *Oscillospira, Prevotella, Helicobacter*, and *Bilophila* reduced, and those of *Jeotgalicoccus, Staphylococcus, Acinetobacter, Aerococcus, Lactobacillus, Corynebacterium, Rikenella, Enterobacter, Proteus, Anaerotruncus*, and *Trabulsiella* increased. These findings indicated a relationship between the gut microbiota and the immune system.

**Figure 6:**
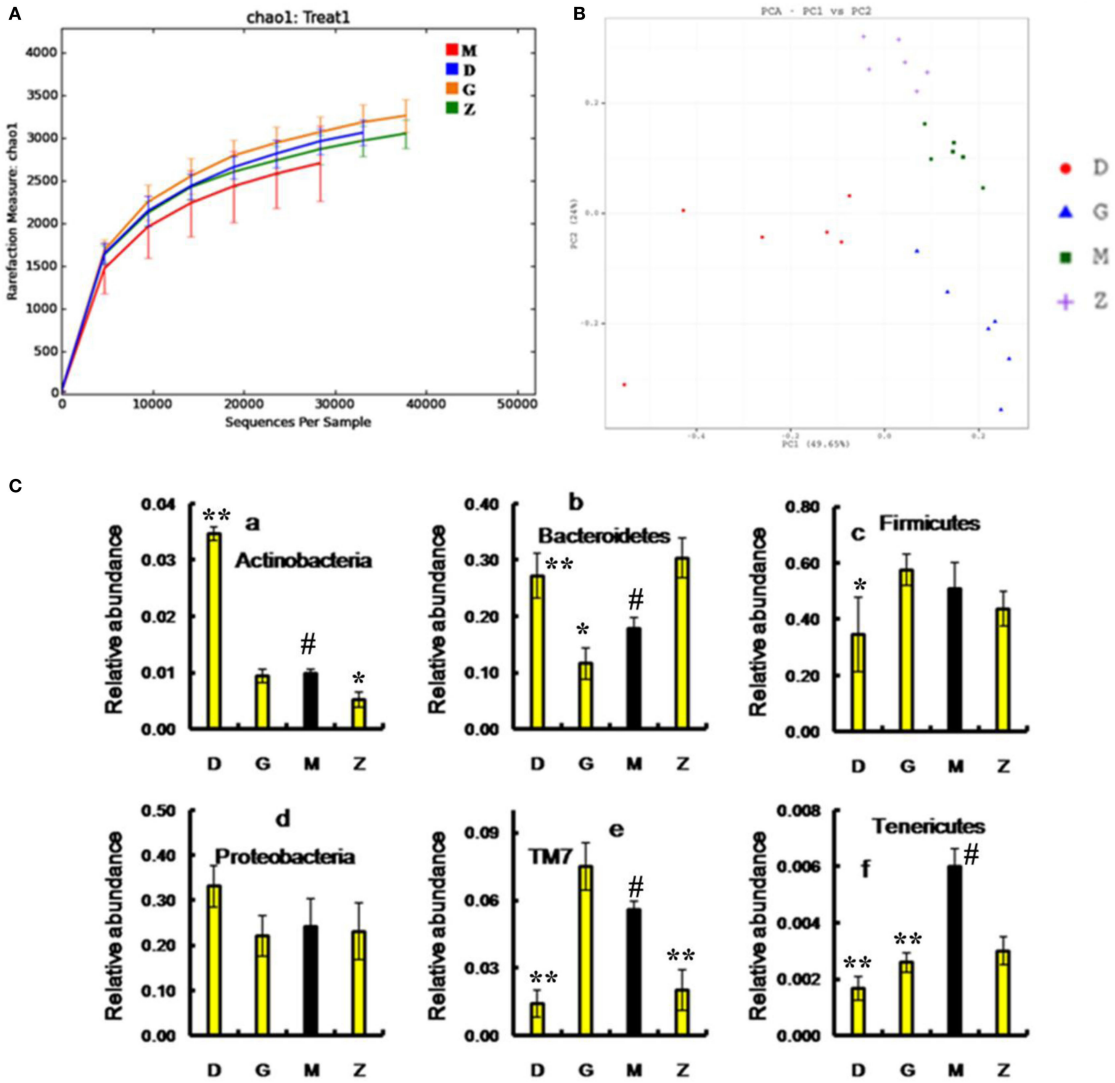

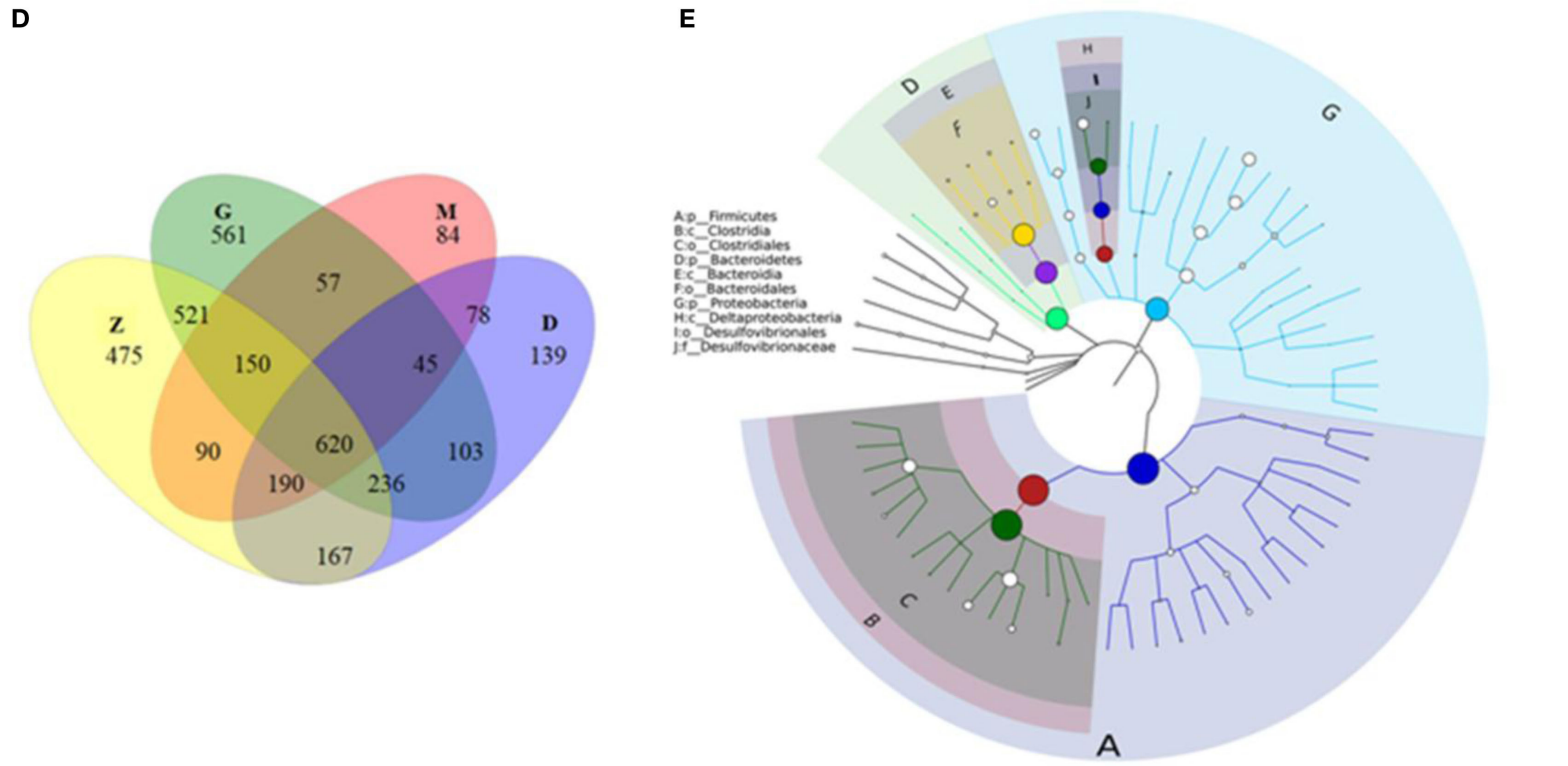
Effects of HEP3 on the microbiota of cecal contents in rats with cyclophosphamide-induced immunotoxicity. **(A)** The rarefaction curve; **(B)** the principal component analysis of operational taxonomic units; **(C)** the classification and abundance of cecal contents at the phylum level; **(D)** the Venn diagram of OTUs; **(E)** the sample species classification tree. Z denotes the control group just treated with vehicle, M is the cyclophosphamide-induced (intraperitoneal injection of 80 mg/kg) group, D is the 100 mg/kg HEP3-treated group, and G is the 200 mg/kg HEP3-treated group. Values were means ± SDs of six independent experiments. ^#^*P* < 0.05 vs the control group, *P < 0.05, **P < 0.01 vs the cyclophosphamide-induced group, indicating significant differences.

**Figure 7:**
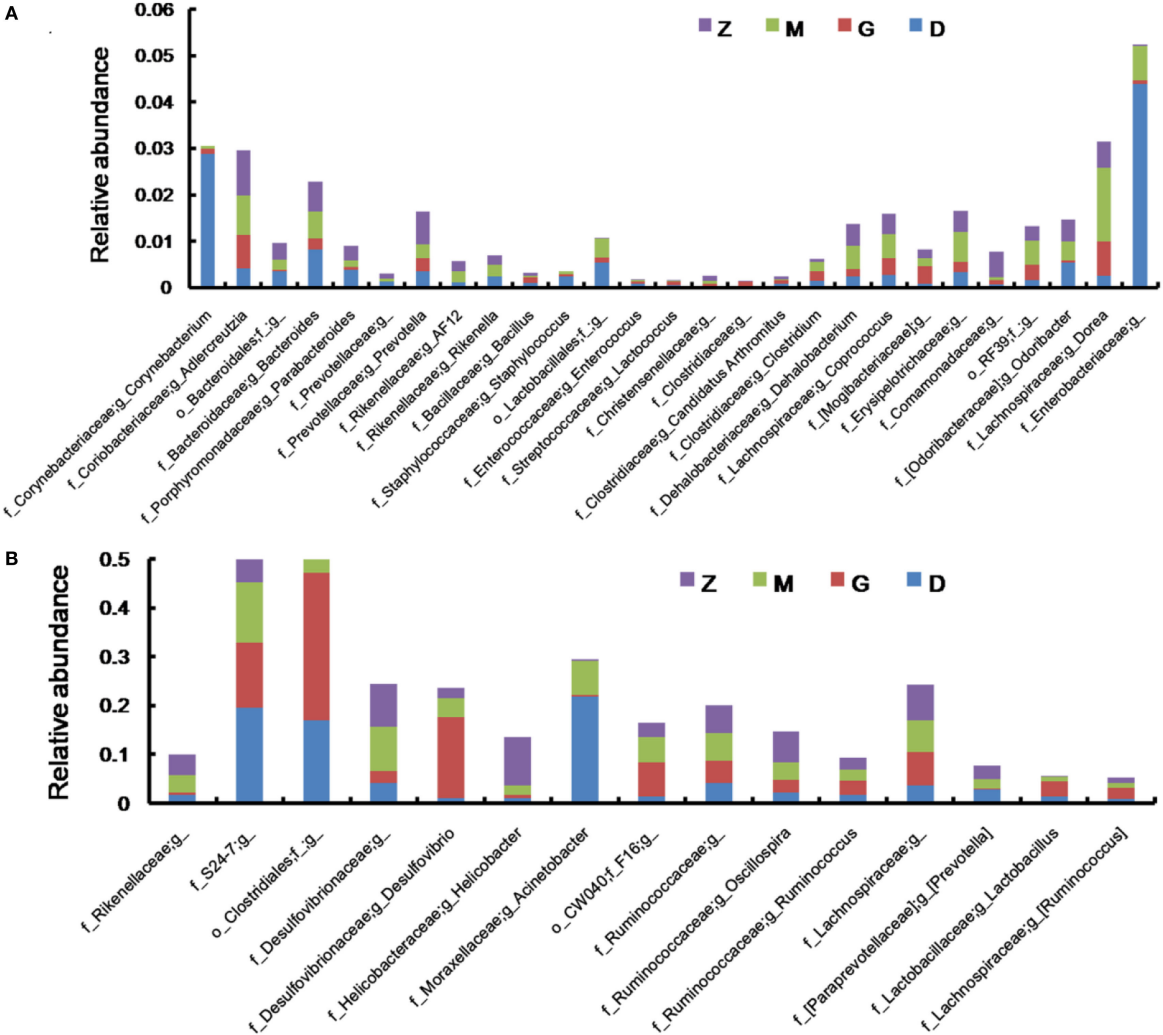

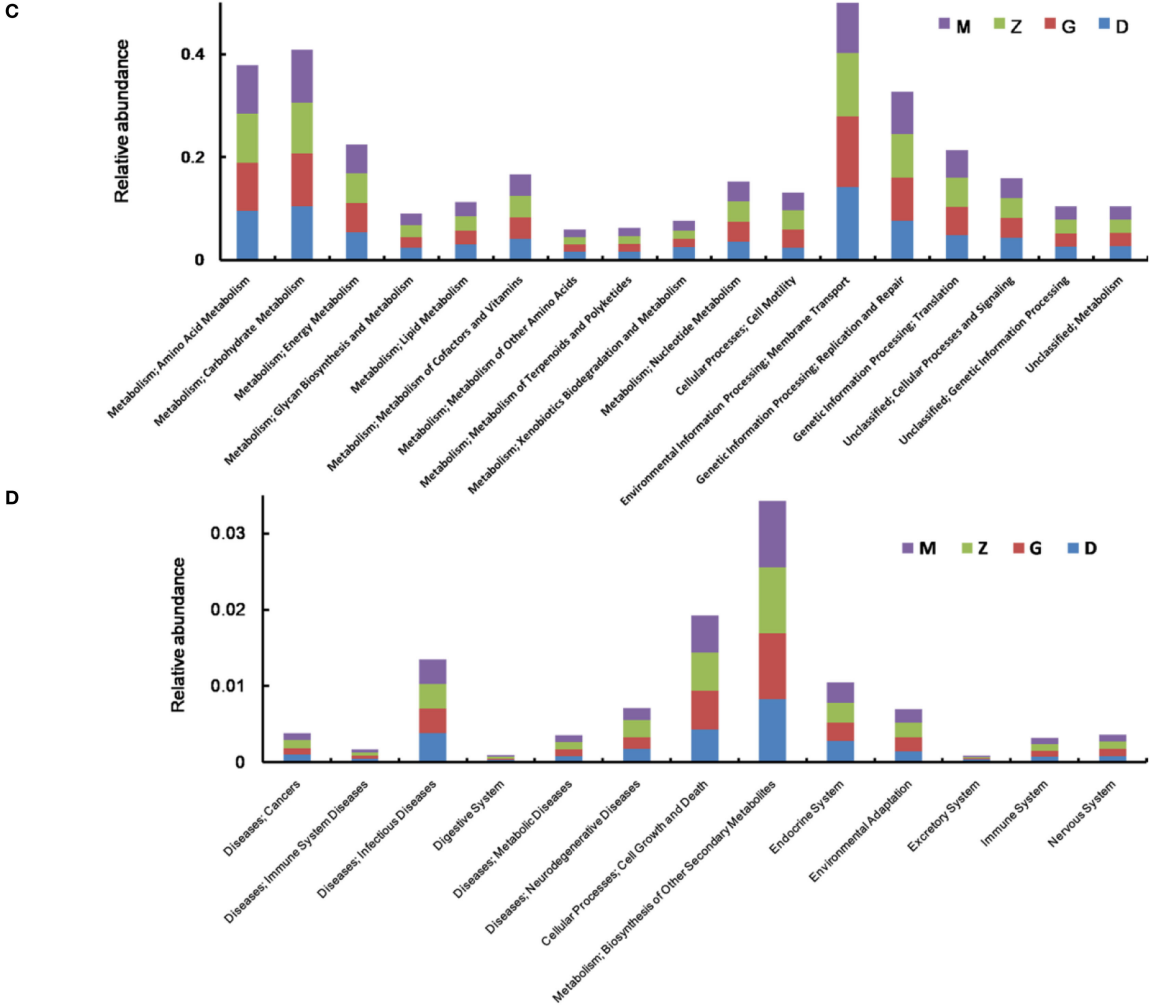
Effect of HEP3 on the microbiota classification and abundance detected by 16S rRNA gene sequencing analysis of cecal contents from the mice with cyclophosphamide-induced immunotoxicity at the genus level **(A,B)**. Effect of HEP3 on KEGG pathways of gut microbiota in mice with cyclophosphamide-induced immunotoxicity **(C,D)**. Z denotes the control group just treated with vehicle, M is the cyclophosphamide-induced (intraperitoneal injection of 80 mg/kg) group, D is the 100 mg/kg HEP3-treated group, and G is the 200 mg/kg HEP3-treated group.

After treatment with HEP3, the gut microbiota was different from that in the high-dose cyclophosphamide-induced and the normal groups (Figure 6). The rarefaction curve (Figure 6A) showed that HEP3 could maintain the diversity of population, as the chao1, ACE, simpson, and shannon of normal group is 3,191.61; 3,266.98; 0.97; 7.45; respectively. The model group was reduced to 2,884.07; 2,974.49; 094; and 6.81, the different were significant by compared to the normal (*P* < 0.05), while these parameters were recovered to 3,303.52; 3,387.48; 0.96; 7.13 for the low-dose HEP3-treated group (*P* < 0.05), and the high-dose group (3,415.14; 3,540.04; 0.96; 7.33; respectively) were better (*P* < 0.01). The PCA (Figure 6B) could successfully distinguish between treatment groups. The cartogram of microbiota at the phylum level is shown in Figure 6C, revealing that the number of *Actinobacteria* (Figure 6C, a), *Tenericutes* (Figure 6C, f), and *TM17* (Figure 6C, e) increased, whereas the number of *Bacteroidetes* (Figure 6C, b) and *Firmicutes* (Figure 6C, c) reduced. In the HEP3-treated group [100 and 200 mg/(kg ⋅ day)], the abundance of *Actinobacteria, Bacteroidetes*, and *Proteobacteria* significantly changed (*P* < 0.05 compared with the high-dose CTX), and was close to the normal (*P* > 0.05). Moreover, the Venn (Figure 6D) results revealed that HEP3 could change the microbiota composition of the cecal contents. The altered diversity of the gut microbiota was also observed at the genus level, as shown in Figure 7. After treatment with HEP3, the diversity of *Corynebacterium, Bacteroides, Enterobacter, Acinetobacter, Desulfovibrio*, and *Lactobacillus* increased, while the abundance of some pathogenic bacteria or conditioned pathogen increased. All the statistical results are shown in Figure 7; the outlier data samples of Z3, M3, and G4 were excluded (Figures 7A,B).

A hierarchical tree was also built using the GraPhlAn software (29), as shown in Figure 6E, revealing that *Firmicutes, Clostridia, Clostridiales, Lachnospiraceae, Bacilli, Lactobacillales, Lactobacillus, Bacteroidetes, Bacteroidia*, and *Bacteroidales* were the advantage groups, which can be used as key researched bacteria while evaluating the immunity of HEP3 in further studies.

The metabolic alterations were analyzed to determine the relationship between the relative abundance of Kyoto Encyclopedia of Genes and Genomes (KEGG) metabolic pathways and immunotoxicity (Figures 7C,D); the metabolism, genetic information processing, and environmental information processing were more or less different. After treatment with CTX, most metabolisms slowed down; while after treatment with HEP3, almost all the characteristic indexes recovered to the normal or were better than that, indicating that HEP3 could balance the metabolic activities of the gut microbiota to maintain the immunity.

### HEP3 Enhanced the Immunity through the Gut Microbiota

#### HEP3 Markedly Relieved the Tissue Damage and Inflammation Induced by TNBS Combined Antibiotics

**Figure 8:**
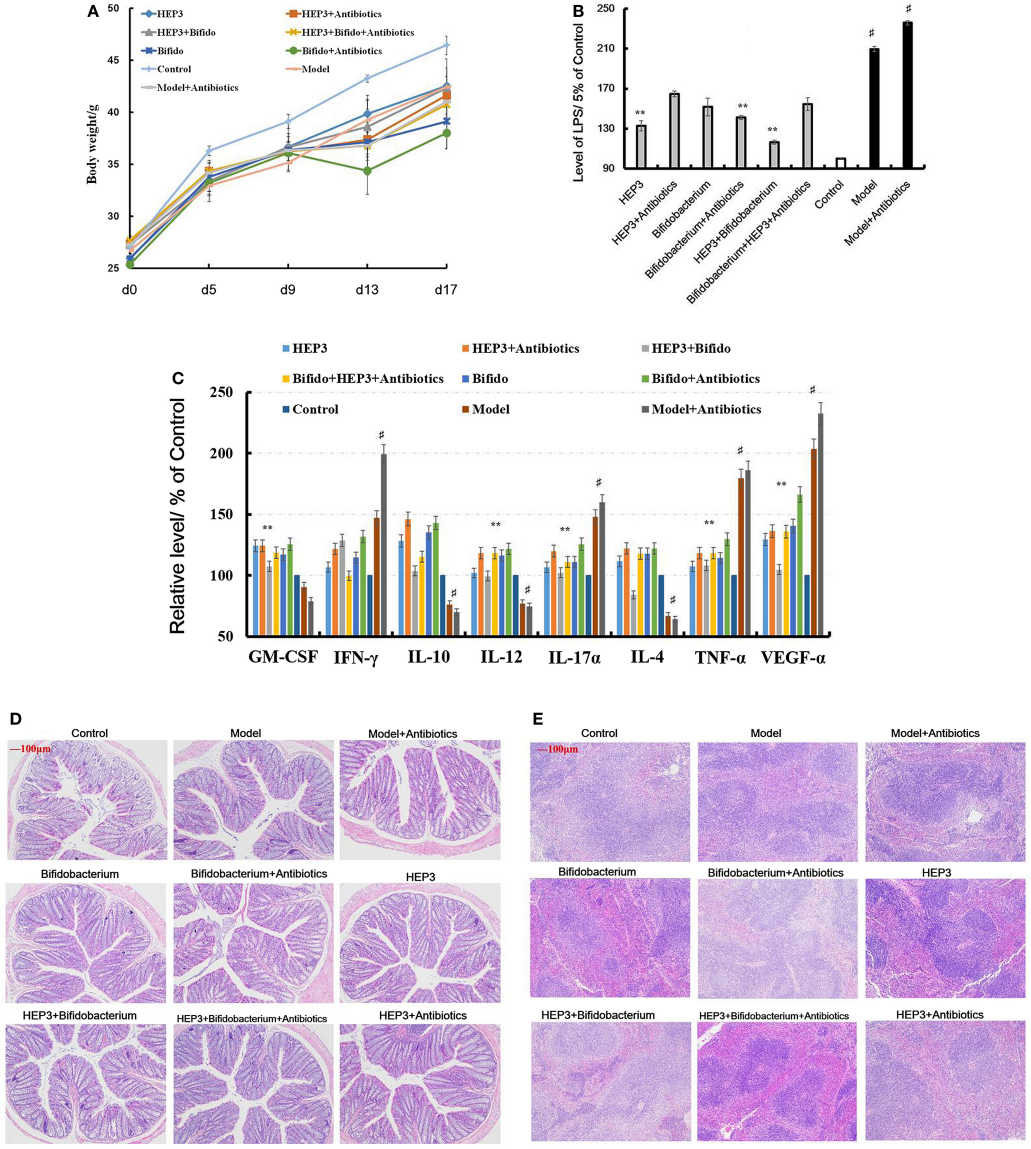
HEP3 extracted from *Hericium erinaceus* improved the pathological parameters of the trinitrobenzenesulfonic acid solution (TNBS)-induced mice. **(A)** The body weight changes; **(B)** the levels of lipopolysaccharide in serum; **(C)** the levels of cytokines GM-CSF, tumor necrosis factor (TNF)-γ, 1L-10, interleukin (IL)-12, 1L-17α, 1L-4, TNF-α, and vascular endothelial growth factor in serum; **(D)** the histopathological changes in colon; and **(E)** the histopathological changes in spleen. Control is the normal group; model is the TNBS-induced group; model and high-dose antibiotics; HEP3 [100 mg/(kg ⋅ day)], *Bifidobacterium*, HEP3 and high-dose antibiotics, HEP3 and *Bifidobacterium, Bifidobacterium* and high-dose antibiotics, HEP3 and *Bifidobacterium* and high-dose antibiotics.

**Figure 9:**
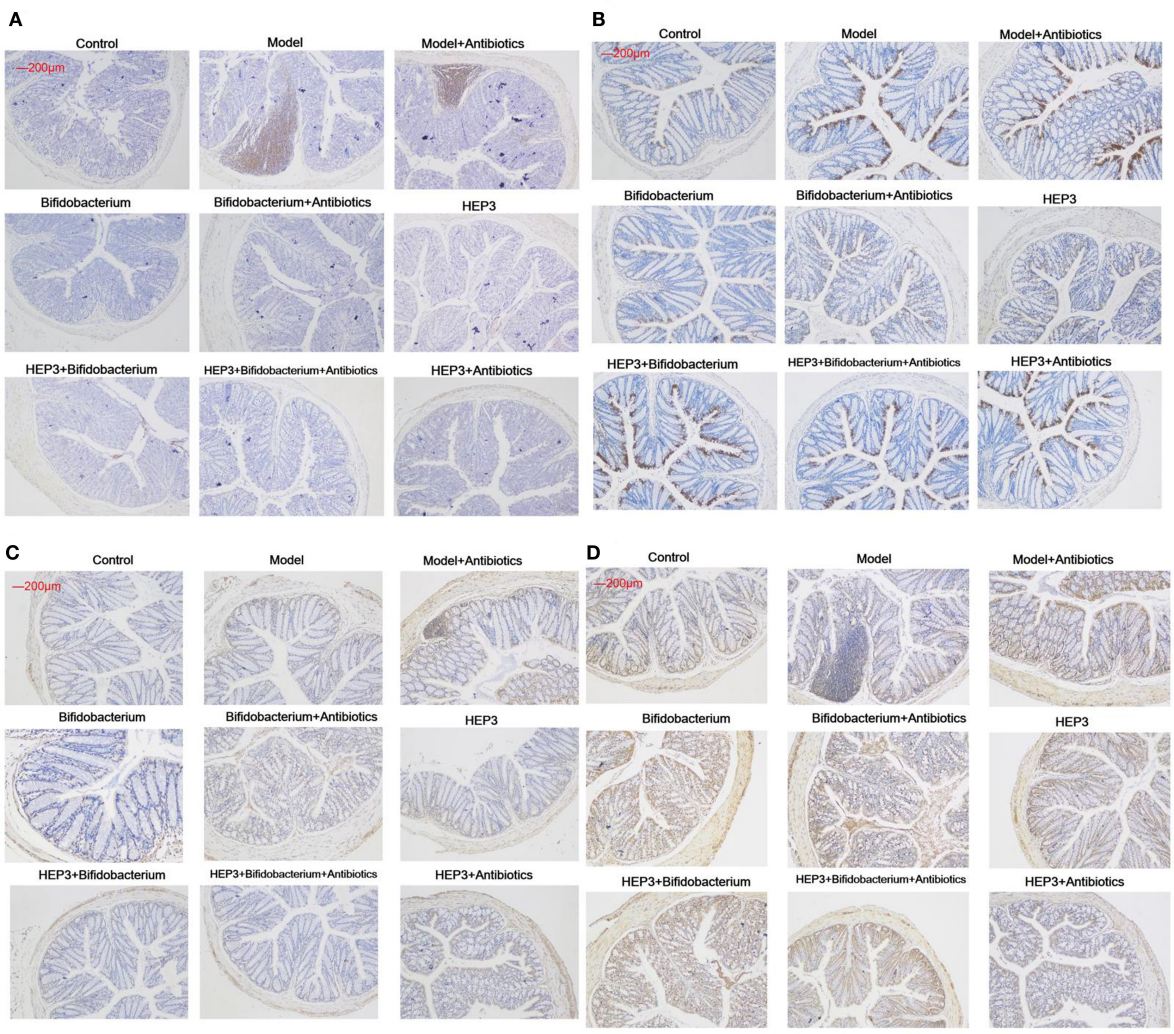
Immunohistochemical staining of tumor necrosis factor-α **(A)**, NF-κB p65 **(B)**, interleukin-17 **(C)**, and Foxp3 **(D)** in the colons of different experimental groups in inflammatory bowel disease mice after treatment with HEP3. Control is the normal group; model is the trinitrobenzenesulfonic acid solution-induced group; model and high-dose antibiotics; HEP3 [100 mg/(kg ⋅ day)], *Bifidobacterium*, HEP3 and high-dose antibiotics, HEP3 and *Bifidobacterium, Bifidobacterium* and high-dose antibiotics, HEP3 and *Bifidobacterium* and high-dose antibiotics.

An IBD mice model was prepared after treatment with broad-spectrum antibiotics, to confirm the relationship between the immunomodulatory activity of HEP3 and gut microbiota. As shown in Figure 8D, the colon tissues were seriously damaged in the TNBS combined antibiotics-treated group compared with those induced by just TNBS, including the splenic tissues (Figure 8E). All the cytokine levels deviated from the normal and TNBS, as some anti-inflammatory cytokines GM-CSF, TNF-γ, 1L-10, IL-12, 1L-17α, 1L-4, TNF-α, and VEGF were secreted significantly differently (*P* < 0.05 or <0.01), as shown in Figure 8C. Meanwhile, the LPS (Figure 8B) levels were higher than those in the TNBS group. These results implied that excess antibiotics resulted in more serious damage and inflammation. After treatment with HEP3, *Bifidobacterium*, and HEP3 + *Bifidobacterium*, all the symptoms and parameters of IBD recovered to near normal, especially in the HEP3 + *Bifidobacterium*-treated group, as shown in Figure 9. Cumulatively, all these results suggested that HEP3 and *Bifidobacterium* had effective anti-inflammatory effects in IBD, and HEP3 and *Bifidobacterium* might act synergistically. However, the mechanism needs further investigation.

#### HEP3 Promoted the Engraftment Ability of *Bifidobacterium* Significantly

**Figure 10:**
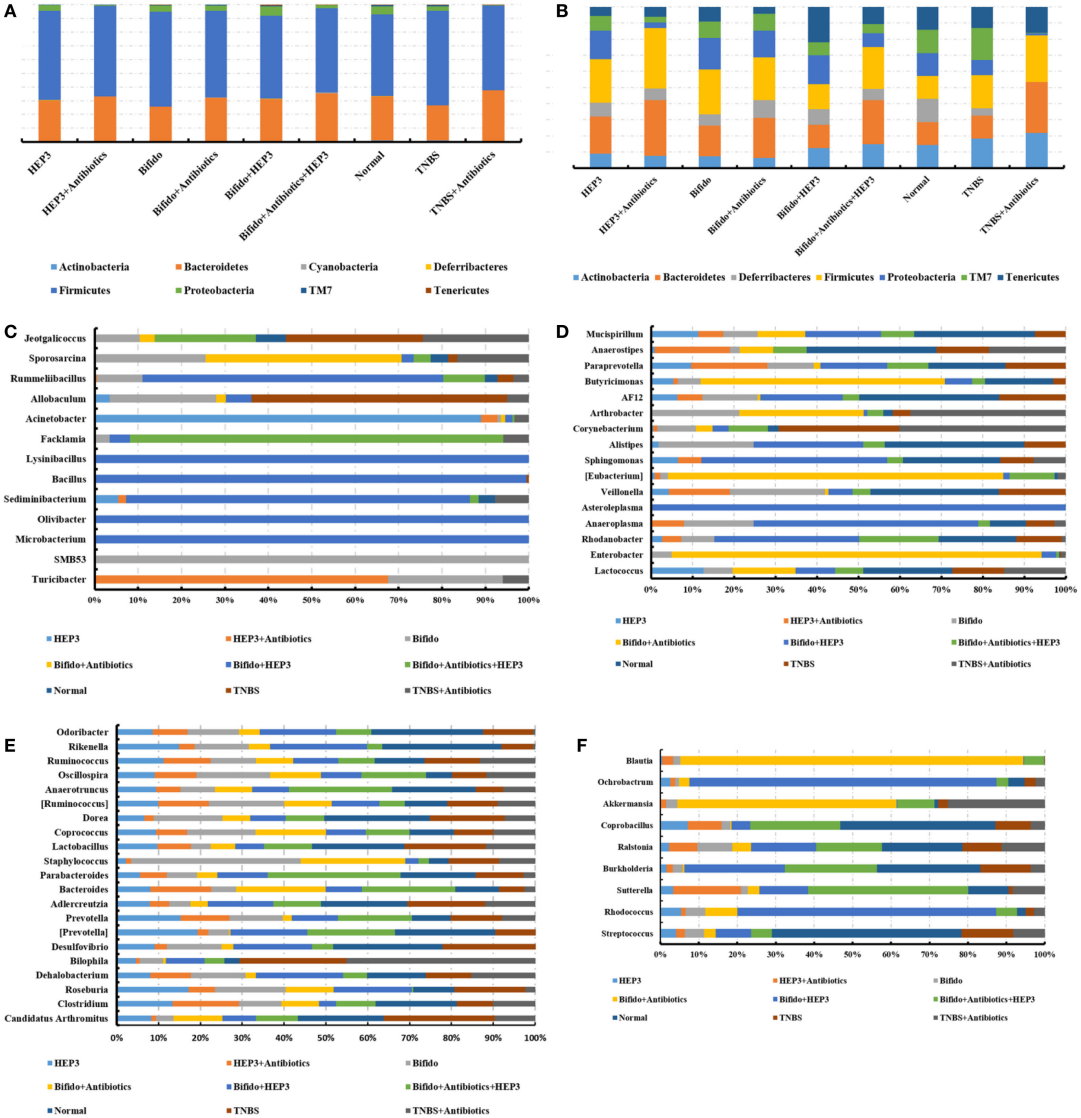
HEP3 showed good prebiotic effects in trinitrobenzenesulfonic acid solution (TNBS)-induced mice. **(A,B)** The relative abundance at the phylum level; **(C–F)** the relative abundance at the family level. Control is the normal group; model is the TNBS-induced group; model and high-dose antibiotics; HEP3 [100 mg/(kg ⋅ day)], *Bifidobacterium*, HEP3 and high-dose antibiotics, HEP3 and *Bifidobacterium, Bifidobacterium* and high-dose antibiotics, HEP3 and *Bifidobacterium* and high-dose antibiotics. Values were means of six independent experiments.

The bacterial composition was analyzed at the genus level to clarify the synergistic action between HEP3 and *Bifidobacterium*, especially the engraftment ability of *Bifidobacterium*. The results showed that the relative abundance of *Bifidobacterium* and other probiotics obviously increased (*P* < 0.05, Figure 10), with more diversity and stable structures. As a result, immunity was significantly enhanced as the expression of TNF-α (Figure 9A), NF-κB (Figure 9B), and IL-17 (Figure 9C) in the HEP3- and *Bifidobacterium-*treated group decreased compared with the model (*P* < 0.05) and TNBS + antibiotics (*P* < 0.01) groups, while the expression of Foxp3 (Figure 9D) increased (*P* < 0.01), indicating that HEP3 could alleviate the high-dose antibiotic-induced destruction of the intestinal microecology and play an effective prebiotic role.

### HEP3 Suppressed Tumor Growth in CC531 Cell Tumor Xenograft Model Mice

**Figure 11:**
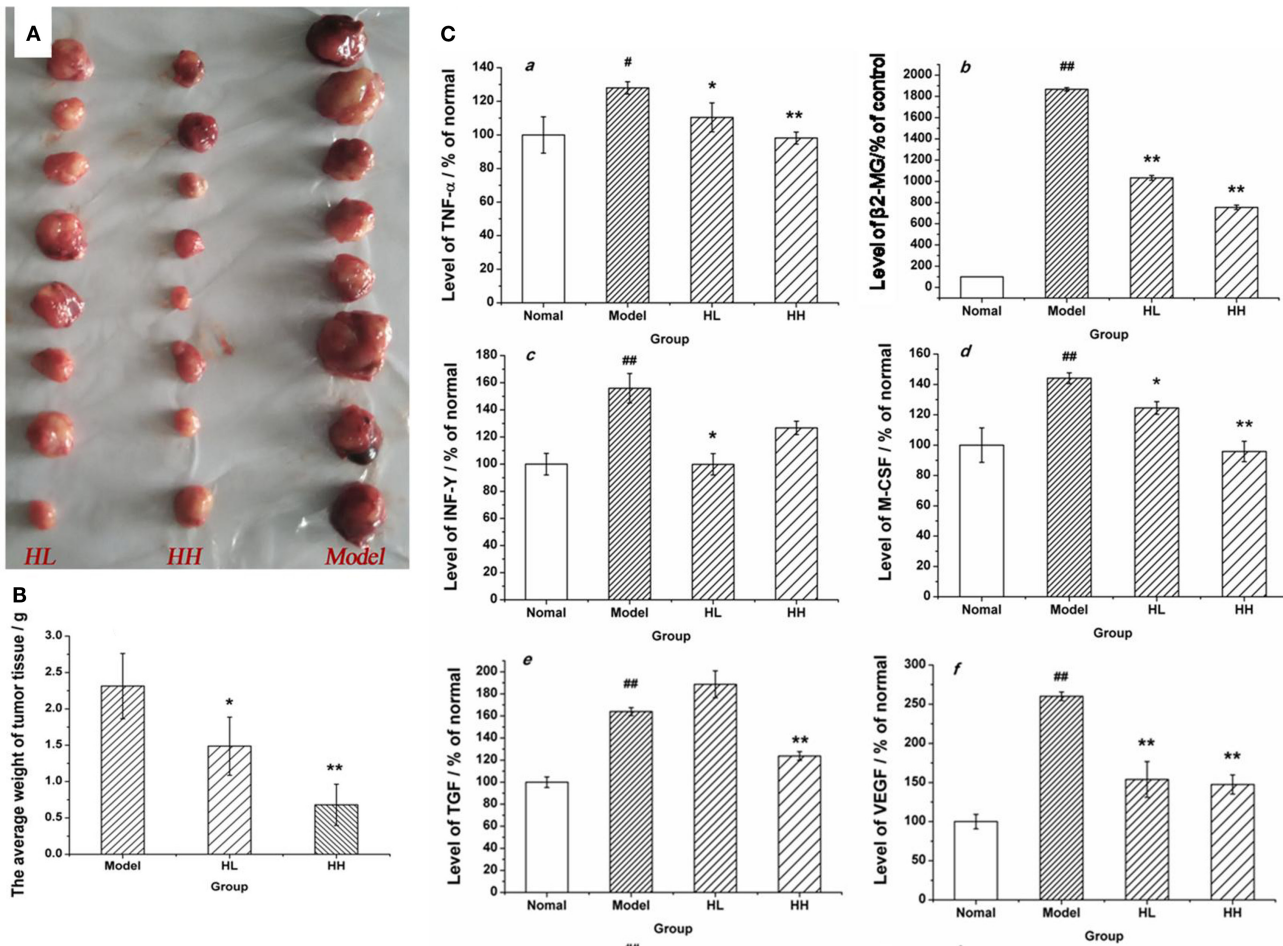
Effects of HEP on the average tumor weight and the content of various cytokines in the CC531 cell tumor xenograft model mice. The tumor tissue of HL, HH, and model groups **(A)**; the tumor inhibition rate **(B)**; the contents of various cytokines **(C)** were detected using an enzyme-linked immunosorbent assay kit: tumor necrosis factor-α (a), PSA (b), interferon-γ (c), macrophage colony-stimulating factor (d), transforming growth factor (e), and vascular endothelial growth factor (f). Values were means ± SDs. ^#^*P* < 0.05, ^##^*P* < 0.01 vs the normal group; *P < 0.05, **P < 0.01 vs the model group, indicating significant differences.

A tumor model was set up in the BALB/c mice by implanting CC531 tumor cells to further prove the immunomodulatory activities of HEP3. After constructing the model, HEP3 was induced through gastric perfusion. Four weeks later, the tumor-related indicators were observed and measured. The mice were sacrificed under ether narcotization after 21 days of treatment. The serum and tumor tissues of mice were used to determine the serum levels of β2-GM and related cytokines TNF-α, IFN-γ, M-CSF, TGF, and VEGF at the same time. The tumor weight of the dose groups was significantly reduced compared with the model group (Figure 11A), and the TIR was calculated. The TIR of the HL and HH groups was 35.73 and 70.61%, respectively, as shown in Figure 11B [TIR = (average tumor weight of the model group − average tumor weight of the experimental group)/average tumor weight of the model group × 100]. The levels of β2-GM, TNF-α, IFN-γ, M-CSF, TGF, and VEGF related to immunity or inflammation improved to near normal (*P* > 0.05), as shown in Figure 11C. All the results demonstrated that HEP3 had a strong inhibitory activity and could be used for the treatment of tumor as a FIP.

## Discussion

The immunomodulatory and antitumor activities of fungal proteins have been widely studied after polysaccharides and terpenoids in recent years (30–34). *H. erinaceus*, as an edible medicinal mushroom, is processed into a variety of products (beverage, cookies, oral liquids, and so on) sold in supermarkets and drugstores. However, all the products are not the proteins of *H. erinaceus*. It is thought that proteins extracted from the fruiting bodies of *H. erinaceus* have immunomodulatory and antitumor properties. A 50- to 55-kDa single-band protein was isolated in this study from the crude protein extracts using alkaline extraction and acid precipitation method, membrane separation technology, and a pharmacodynamic evaluation method. Evaluations revealed that HEP3 had a strong anti-inflammatory and immunohypofunction and could be used for treating IBD, hypoimmunity, or even tumors.

Immune factors play a predominant role in the pathogenesis of IBD (35, 36). Cytokines, including 1L-1, 1L-2, IL-12, TNF-α, VEGF, and MIP-α, are proinflammatory, while 1L-8, 1L-10, 1L-11, TNF-γ, and M-CSF are anti-inflammatory. These cytokines have many biological activities as transfer molecules, mainly regulating immune response, participating in the immune cell differentiation development and tissue repair, interfacing inflammation, and stimulating hematopoietic function and other functions. Anti-inflammatory and immunosuppressive treatments reduce and limit the damage caused by IBD (37). The evaluation tests showed that HEP had a strong anti-inflammatory activity in IBD model rats and mice, indicating that HEP was a functional food ingredient for immunoregulation. Further, a single-band protein was isolated (HEP3) using the membrane separation technology, and RAW 264.7 macrophages were employed to evaluate the immunomodulatory activities. The results revealed that HEP3 elicited strong responses to TNF-α, 1L-1β, and 1L-6. It also suppressed the LPS-induced production of inflammatory cytokines in the RAW 264.7 macrophages through suppressing NF-κB DNA-binding activity, followed by the downregulation of iNOS activity, eventually resulting in the decrease in NO production. However, the detailed molecular mechanism and characteristics need to be revealed in further studies.

Growing empirical evidences have shown that the diversity of gut microbiota in IBD patients is reduced (38, 39). The most consistent observations of altered composition of the gut microbiota in IBD patients are a reduction in *Firmicutes* and an increase in *Proteobacteria*, which were same as in the cyclophosphamide-induced mice (Figure 5). In this study, after treatment with 80 mg/(kg ⋅ day) of cyclophosphamide for 4 days, the composition of the cecal content microbiota changed significantly compared with the normal group, as shown in Figures 5 and 6, revealing that the gut microbiome plays an important role in immune regulation and host defense. Previous studies have demonstrated that the gut microbiota have a barrier function to protect the host from the intestinal pathogen attacks (40) and immune regulation functions by regulating the proliferation and differentiation of T cells, and stimulating the intestinal antigen-presenting cells and some bacteria active metabolites (41, 42).

Previous studies have shown that the efficacy of the anticancer immunomodulatory agent CTX relies on intestinal bacteria (43, 44), and high doses often damage the intestinal mucosa and metabolism, and probiotic bacteria such as *Lactobacillus* and *Bifidobacterium* can reduce intestinal mucosal injury and improve intestinal metabolism and intestinal microbiota (45, 46). In this study, CD3^+^, CD4^+^, CD8^+^, CD28^+^, and naive T cells were inhibited in high-dose cyclophosphamide-induced immunotoxicity mice after treatment with HEP3 (Figure 4), and also the immunohistochemistry of colon tissues in the IBD model rats showed the same results that Foxp3, IL-10, TNF-α, and NF-κB p65 improved to near normal (Figures 1C and 9), indicating that HEP3 might improve the immune function *via* regulating the proliferation and differentiation of T cells with the help of gut microbiota, but much more details need to be revealed.

The IBD model mice were prepared by TNBS enema after treatment with a large range of broad-spectrum antibiotics to explore if the gut microbiota took part in immunity activated by HEP3. As shown in Figures 8–10, without the microbiota, the colon tissues were easily damaged and inflamed (antibiotics-treated groups) compared with the only TNBS-induced group, which verified that gut microbiota could make an intestinal mucous membrane surface to form a biological barrier. With the help of HEP3, the *Bifidobacterium* abundance increased significantly (*P* < 0.05), and also the colon tissue damages, inflammation, other prebiotics, and diversity and structures improved significantly. These results confirmed that HEP3 had immunomodulatory activities and could serve as a good prebiotic.

**Figure 12:**
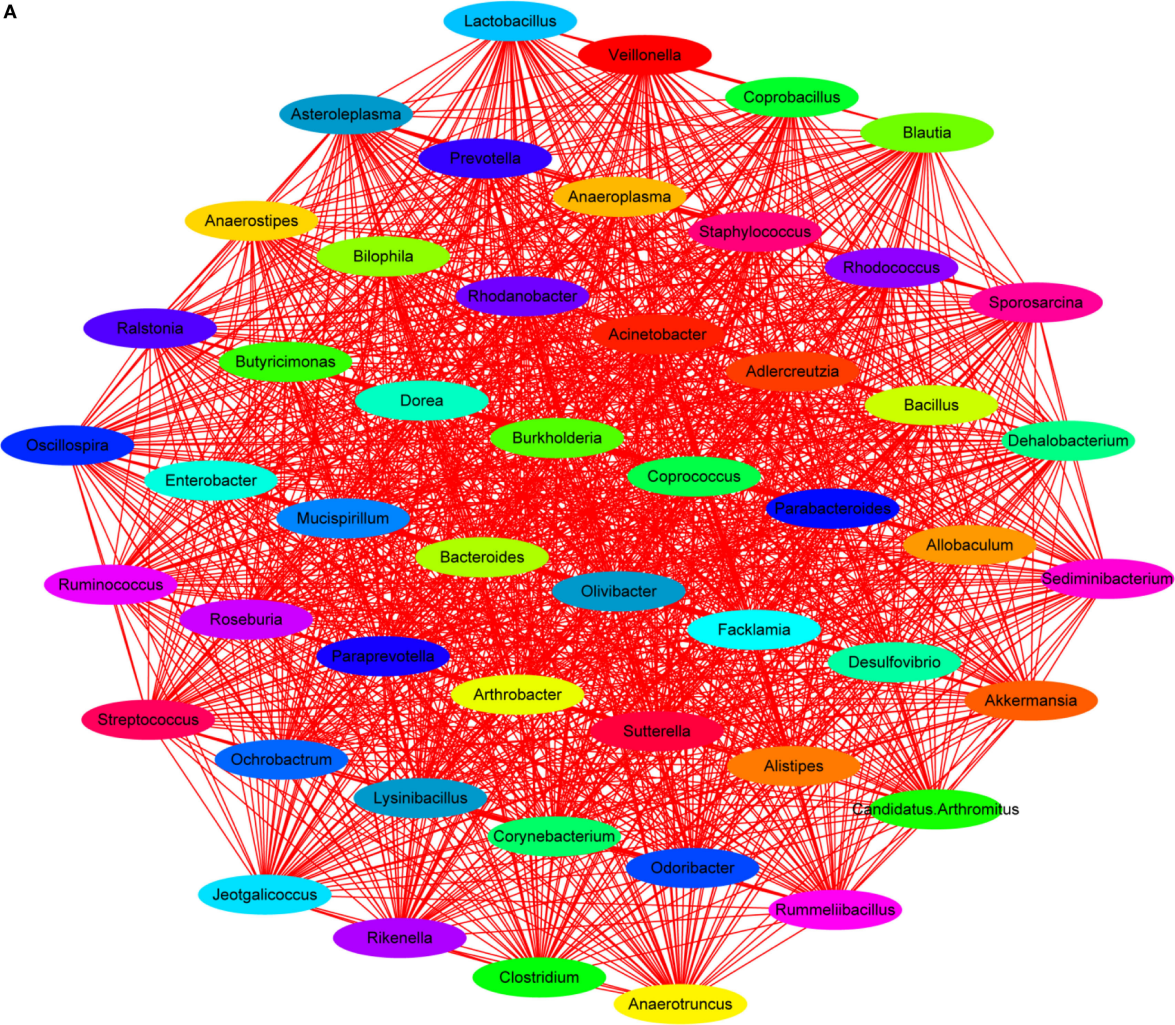

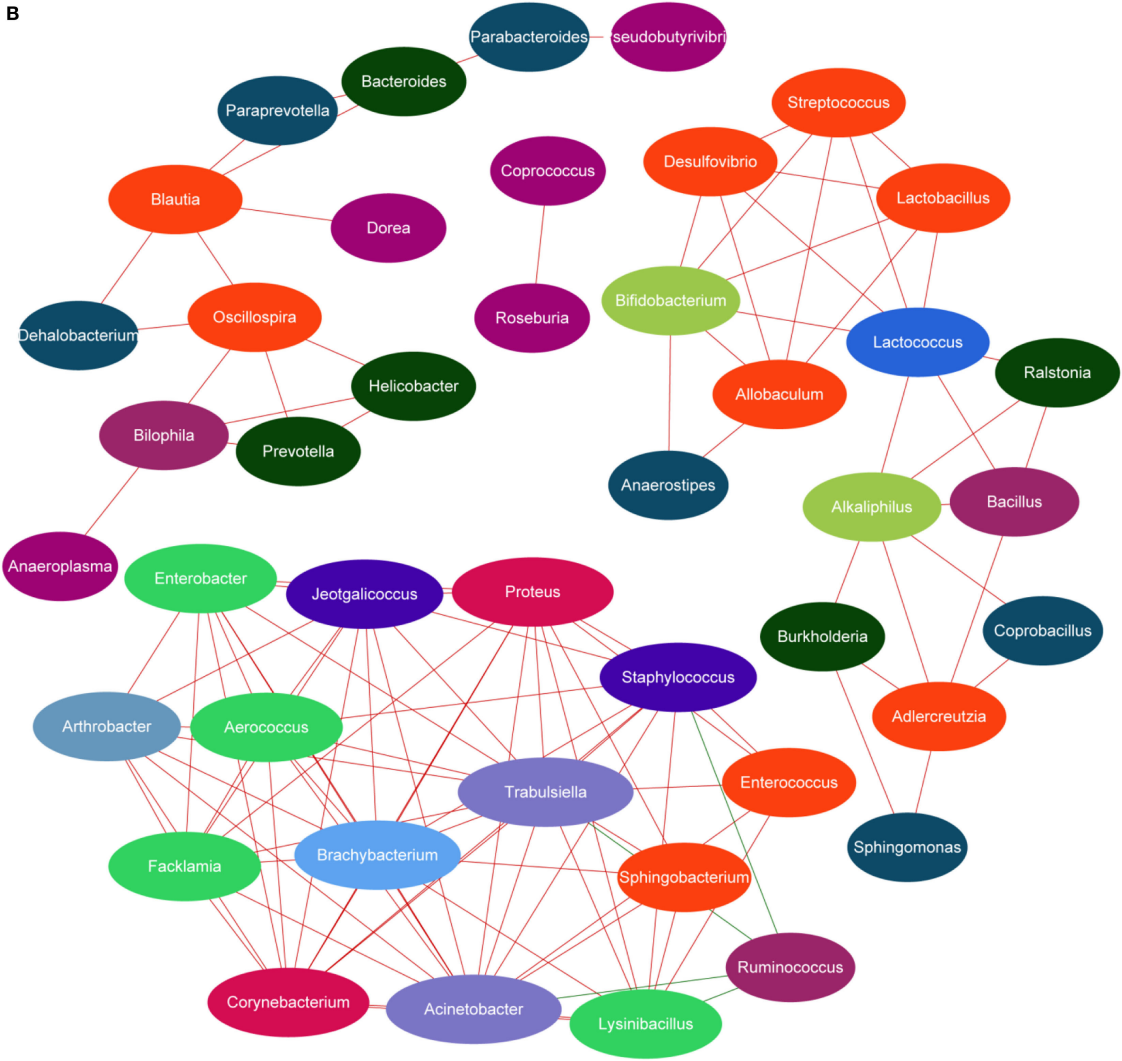
Dominant species interaction-associated network analysis results. Nodes represent the dominant species under the identity of different colors: the red line shows a positive correlation and the green line shows a negative correlation. More connections by nodes indicated more association with others. **(A)** Antibiotics rapidly declined the diversity and destroyed the stability of the whole ecological system; **(B)** HEP3 can main the diversity and stability of the whole ecological system in IBD model mice.

Lipopolysaccharide, mainly secreted from *Bacteroides* spp., *B. vulgatus*, and *Desulfovibrio* spp. (45, 46), is regarded as a stimulating factor for inflammation (Figure 8B). In this study, the levels of LPS were reduced after treatment with HEP3 and *Bifidobacterium*, and the abundance of *Bacteroides* spp., *B. vulgatus*, and *Desulfovibrio* spp. decreased, revealing that HEP3 inhibited the proliferation of these bacteria and hence reduced the secretion of LPS. How HEP3 influences the proliferation of *Bacteroides* spp., *B. vulgatus*, and *Desulfovibrio* spp. needs further exploration. This study also found that antibiotics rapidly declined the diversity and destroyed the stability of the whole ecological system (Figure 12A). Some special foods might help in controlling this situation (Figure 12B). These results were consistent with previous reports that antibiotics were the most influencing factors on gut microbiota (47).

HEP3 is a protein, and its digestion and absorption need many proteases and peptidases extracted from bacteria. In contrast, proteins and their degradation products serve as important nitrogen sources and growth factors, or even energy sources, for some anaerobic organisms (48). Therefore, HEP3 can significantly influence the diversity, structures, and metabolism of organisms and microorganisms. As shown in Figures 7 and 10, the diversity and structures were recovered with the treatment of HEP3, and some metabolic pathways were reactivated to near normal. Besides the improvement in IBD rats and mice, this study concluded that the changes in the gut microbiota structure might not be consistent in the high-dose cyclophosphamide-induced mice but could improve the disease, and that a steady gut microbiota was extremely important for health.

The aging of intestinal mucosa cells is one of the reasons for inflammation and immunotoxicity. d-galactose, which is a reducing sugar, could induce senescence in the cells of rodents through the overproduction of reactive oxygen species and advanced glycation end products (49–51). The antiaging ability plays an important role in maintaining the immune system (52) and protecting from all living organisms from inflammation. In this study, HEP obviously reversed the d-galactose-induced oxidative stress (increased the GSH-Px and SOD levels, while reducing the MDA level) in the HIEpiC cells, implying that the antiaging activity was also an impetus for enhancing the immunity.

Mushrooms produce many bioactive proteins, including FIPs, ribosome-inactivating proteins, lectins, ribonucleases, antibacterial/antifungal proteins, laccases, and other proteins (32, 53, 54). Although increasing reports are available on the isolation, purification, and functions of mushroom proteins, the mechanisms of their actions (e.g., immunomodulation, antiproliferation, antivirus, antimicrobes, etc.) are still poorly understood. Therefore, novel technologies should be promising in this aspect, and the relationship between structure and bioactivity should be considered.

In summary, a single-band protein (HEP3) isolated from HEP exhibited immunomodulatory activities and could be used as a drug or functional food ingredient for immunotherapy in gastrointestinal diseases. Moreover, HEP3 could improve the immune system *via* regulating the composition and metabolism of gut microbiota to activate the proliferation and differentiation of T cells, stimulate the intestinal antigen-presenting cells, and hence play a prebiotic role.

## Author Contributions

CD, ZC, YJ, LJ, SJ, XY, and LG conceived and designed the experiments. CD, ZC, YJ, LJ, SJ, and LG performed the experiments. CD, ZC, and YJ analyzed the data. CD and ZC wrote the paper and edited the manuscript. All authors read and approved the final manuscript.

## Conflict of Interest Statement

The authors declare that the research was conducted in the absence of any commercial or financial relationships that could be construed as a potential conflict of interest.
