# Supplementary material for: Immunomodulatory Activities of a Fungal Protein Extracted from Hericium erinaceus through Regulating the Gut Microbiota
Source: Front Immunol. 2017 Jun 12;8:666. doi: 10.3389/fimmu.2017.00666 (PMC5492111; doi:10.3389/fimmu.2017.00666)
Supplement: Supplementary file 1 [file Presentation_1.pdf]

**Supplemental materials**

**1 Preparation of the HEP**

A single factor design was carried out to investigate the effect of the variables on the response: the variables investigated were pH of the extraction solvent (7, 8, 9, 10, and 12, respectively), ratio water to raw material (RWM, 10, 20, 30, 40 and 50 ml/g, respectively), extraction time (ETi, 1, 2, 4 and 6 h, respectively), extraction temperature (ETe, 30, 35, 45, 55 and 65 °C, respectively). Protein, soluble in PBS, adjust pH to neutral. The content of protein was detected by Coomassie brilliant blue G-250 methods [29], and the experiments were performed in random order and at least triplicate.

A single factor test was employed to determine the preliminary range of the extraction variables including $X_1$ (pH of extraction solvent), $X_2$ (Ratio water to raw material), $X_3$ (Extraction temperature) and $X_4$ (Extraction time). Then, a three-level-four-factor Box-Behnken factorial design (BBD) (Design Expert software, Trial Version 8.0.5b, Stat-Ease Inc., Minneapolis, MN, USA) was applied to determine the best combination of extraction variables for the yields of water-soluble protein in *H. erinaceus,* as shown in Supp.Tab.1.

**Supp.Tab.1.** Independent variables and their levels used for Box-Behnken design (BBD)

| Independent variables | Symbol | Levels | | |
|---|---|---|---|---|
| | | -1 | 0 | 1 |
| pH | $X_1$ | 7 | 9 | 11 |
| Ratio water to raw material/(ml/g) | $X_2$ | 20 | 35 | 50 |
| Extraction temperature (°C) | $X_3$ | 30 | 40 | 60 |
| Extraction time (min) | $X_4$ | 2 | 4 | 6 |

Experimental data were fitted to a quadratic polynomial model and regression coefficient obtained. The non-linear computer-generated quadratic model used in the response surface was as follows:

$$Y=\beta_0+\sum_{j=1}^{k}\beta j Xj+\sum_{j=1}^{k}\beta jj Xj^2+\sum\sum_{i<j}\beta ij XiXj$$

Where $Y$ is the estimated response, $\beta_0$, $\beta_j$, $\beta_{jj}$ *and* $\beta_{ij}$ are the regression coefficients for intercept, linearity, square and interaction, respectively, while $X_i$, $X_j$ are the independent coded variables. Design Expert software was used to estimate the response of each set of experimental design and optimized conditions. The fitness of the polynomial model equation was expressed by the coefficient $R^2$. $F$-test and $p$-value were used to check the significance of the regression coefficient. Data were expressed as the means (SEM) of three replicated determinations.

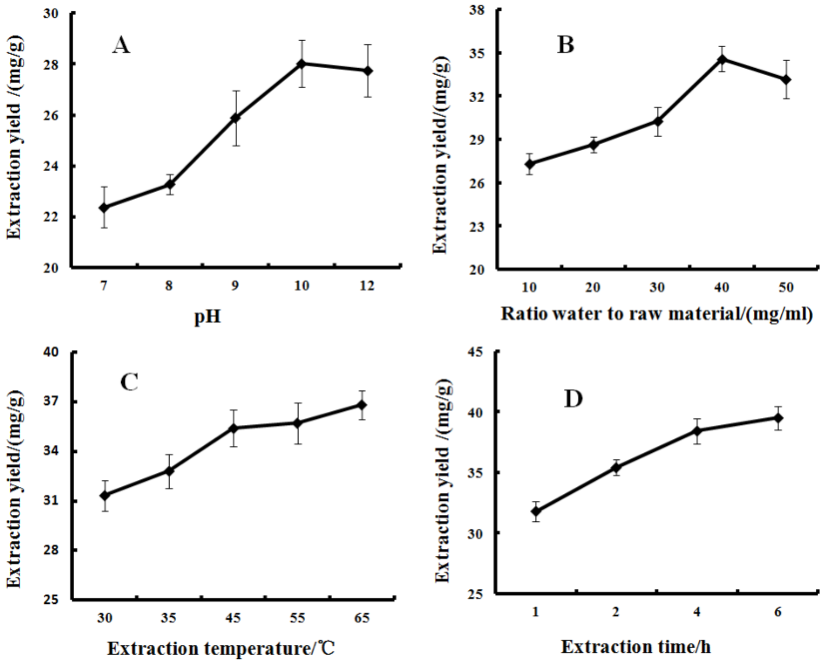


**Supp.Fig.1.** Effects of RWM, ETe, ETi and ETis on the water-soluble protein yield. Ratio of water to raw material (RWM), Extraction time (ETi), Extraction temperature (ETe)

## 2 Single factor test results

In order to obtain the main compositional parameters of the water-soluble protein extraction, several parameters were investigated. According to the analytical results in Supp.Fig.1, the effects of pH and RWM on the water-soluble protein yield were more obviously than the other two factors.

**3 Box-Behnken factorial design results and analysis**

The experimental design and corresponding response data of which, shown in Supp.Tab.2, were determined according to the results of preliminary experiments.

**Supp.Tab.2.** The Box-Behnken design matrix and the results for extraction yield of *HEP*

| Run | $X_1$ (pH) | $X_2$ (Ratio of water to raw material, ml/g) | $X_3$ (Extraction temperature, °C) | $X_4$ (Extraction time, h) | Yield (mg/g) |
|---|---|---|---|---|---|
| 1 | 11 | 35 | 60 | 4 | 25.10 |
| 2 | 9 | 20 | 45 | 6 | 28.27 |
| 3 | 7 | 35 | 60 | 4 | 16.28 |
| 4 | 7 | 50 | 45 | 4 | 18.56 |
| 5 | 11 | 35 | 45 | 2 | 24.00 |
| 6 | 9 | 35 | 60 | 6 | 28.05 |
| 7 | 9 | 35 | 30 | 6 | 29.93 |
| 8 | 9 | 20 | 30 | 4 | 28.86 |
| 9 | 7 | 35 | 30 | 4 | 12.06 |
| 10 | 9 | 35 | 45 | 4 | 29.80 |
| 11 | 11 | 50 | 45 | 4 | 24.47 |
| 12 | 9 | 50 | 30 | 4 | 29.35 |
| 13 | 9 | 20 | 60 | 4 | 27.48 |
| 14 | 11 | 35 | 45 | 6 | 25.54 |
| 15 | 9 | 35 | 45 | 4 | 29.69 |
| 16 | 7 | 20 | 45 | 4 | 12.64 |
| 17 | 9 | 35 | 45 | 4 | 29.90 |
| 18 | 9 | 20 | 45 | 2 | 28.07 |
| 19 | 9 | 35 | 45 | 4 | 29.81 |
| 20 | 11 | 35 | 30 | 4 | 25.65 |
| 21 | 9 | 50 | 45 | 2 | 29.38 |
| 22 | 9 | 35 | 45 | 4 | 29.83 |
| 23 | 7 | 35 | 45 | 6 | 14.88 |
| 24 | 9 | 50 | 60 | 4 | 27.75 |
| 25 | 9 | 35 | 60 | 2 | 27.54 |
| 26 | 11 | 20 | 45 | 4 | 24.91 |
| 27 | 9 | 50 | 45 | 6 | 31.83 |
| 28 | 9 | 35 | 30 | 2 | 27.11 |
| 29 | 7 | 35 | 45 | 2 | 14.67 |

Supp.Tab.3 showed the analysis of variance (ANOVA) for the extraction yield of the water-soluble protein

using response surface methodology. The determination coefficient ($R^2$) of the model is 0.9860, with significant lack of fit at $p$<0.05. That means that the calculated model was able to explain 98.60% of results. The results indicated that the model used to fit responses variable was significant ($p$<0.01) and adequate to represent the relationship between the responses and the independent variables. The $F$-value, 70.382 implied that the model was highly significant. The adjusted determination coefficient ($R^2_{adj}$) of 0.9720 indicated that 3.00% of the total variations cannot be explained by the calculated model. Meanwhile, the coefficient of variation (C.V. %=0.88) indicated that the model was reproducible.

Supp.Tab.3 showed that the water-soluble protein extraction yield was affected significantly ($p$<0.01). The predicted second-order polynomial model was:

$$Y=-228.491+47.797X_1+0.544X_2+0.848X_3+0.806X_4-0.0531X_1X_2-0.0398X_1X_3$$
$$+0.0829X_1X_4-.0.000231X_2X_3+0.0187X_2X_4-0.0192X_3X_4-2.331X_1^2$$
$$-0.000984X_2^2-0.00455X_3^2-0.127X_4^2 \qquad (2)$$

The significance of each coefficient was also determined using $F$-value and $p$-value. The results were given in Supp.Tab.3. It could be seen that the extraction yield was affected significantly by four extraction parameters ($X_1$, $X_2$, $X_4$, $p$<0.05). In addition, it was evident that the quadratic parameters ($X_1^2$, $X_3^2$, $X_1X_2$ and $X_1X_3$) were significant at the level of $p$<0.05, whereas the quadratic parameters ($X_3$, $X_2^2$, $X_4^2$ , $X_1X_2$, $X_2X_3$, $X_2X_4$ and $X_3X_4$) were insignificant ($p$>0.05).

To determine optimal levels of the variables for the water-soluble protein extraction, the contour plots and three-dimensional surface plots were constructed according to equation. Supp.Figs 2 and 3 showed the effects of the independent variables and their mutual interaction on the extraction yield of water-soluble protein. The intuitive result from two figures showed that the effect of four extraction parameters on the yield of

water-soluble protein were significant.

**Supp.Tab.3.** Analysis of variance (ANOVA) for response surface quadratic model

| **Source** | **Sum of Squares** | ***df*** | **Mean Square** | ***F*-Value** | **Prob > *F*** |
|---|---|---|---|---|---|
| Model | 927.836 | 14 | 66.274 | 70.382 | < 0.0001 |
| $X_1$ | 305.873 | 1 | 305.873 | 324.834 | < 0.0001 |
| $X_2$ | 10.293 | 1 | 10.293 | 10.931 | 0.0052 |
| $X_3$ | 0.048 | 1 | 0.048 | 0.051 | 0.8252 |
| $X_4$ | 4.949 | 1 | 4.949 | 5.256 | 0.0379 |
| $X_1X_2$ | 10.148 | 1 | 10.148 | 10.777 | 0.0054 |
| $X_1X_3$ | 5.695 | 1 | 5.695 | 6.048 | 0.0275 |
| $X_1X_4$ | 0.440 | 1 | 0.440 | 0.467 | 0.5053 |
| $X_2X_3$ | 0.011 | 1 | 0.011 | 0.011 | 0.9163 |
| $X_2X_4$ | 1.254 | 1 | 1.254 | 1.332 | 0.2678 |
| $X_3X_4$ | 1.331 | 1 | 1.331 | 1.413 | 0.2543 |
| $X_1^2$ | 563.870 | 1 | 563.870 | 598.823 | < 0.0001 |
| $X_2^2$ | 0.318 | 1 | 0.318 | 0.338 | 0.5704 |
| $X_3^2$ | 6.799 | 1 | 6.799 | 7.220 | 0.0177 |
| $\mathrm{X}_4^2$ | 1.685 | 1 | 1.685 | 1.789 | 0.2023 |
| Residual | 13.183 | 14 | 0.942 | | |
| Lack of Fit | 13.161 | 10 | 1.316 | 244.015 | < 0.0001 |
| Pure Error | 1.077 | 4 | 0.005 | | |
| Cor Total | 624.585 | 28 | | | |
| $R^2$ | 0.9860 | | Adep precision | 26.523 | |
| $R^2_{adj}$ | 0.9720 | | C.V.% | 3.85 | |

In the present investigation, the software predicted that the optimum pH, ratio of water to raw materials, extraction temperature and extraction time were 9.47, 50.00, 37.88, and 6.00, respectively. The software predicted the optimized extraction yield of water-soluble protein to be 31.81 mg/g, while three parallel experiments which were carried out under the optimal conditions, in which the average extraction yield of water-soluble protein 30.10±0.93 mg/g. Compared with the value predicted by Design-Expert 8.0.5b, the results showed that the predicted value was very close to the actual result, indicating that the optimization parameters proposed are reliable. So the optimal extraction conditions for the bioactive compounds were as

follows: 9.47 of pH, 50 ml/g of ratio of water to raw materials, 6 h of extraction time, and 37.9 °C of extractive temperature.

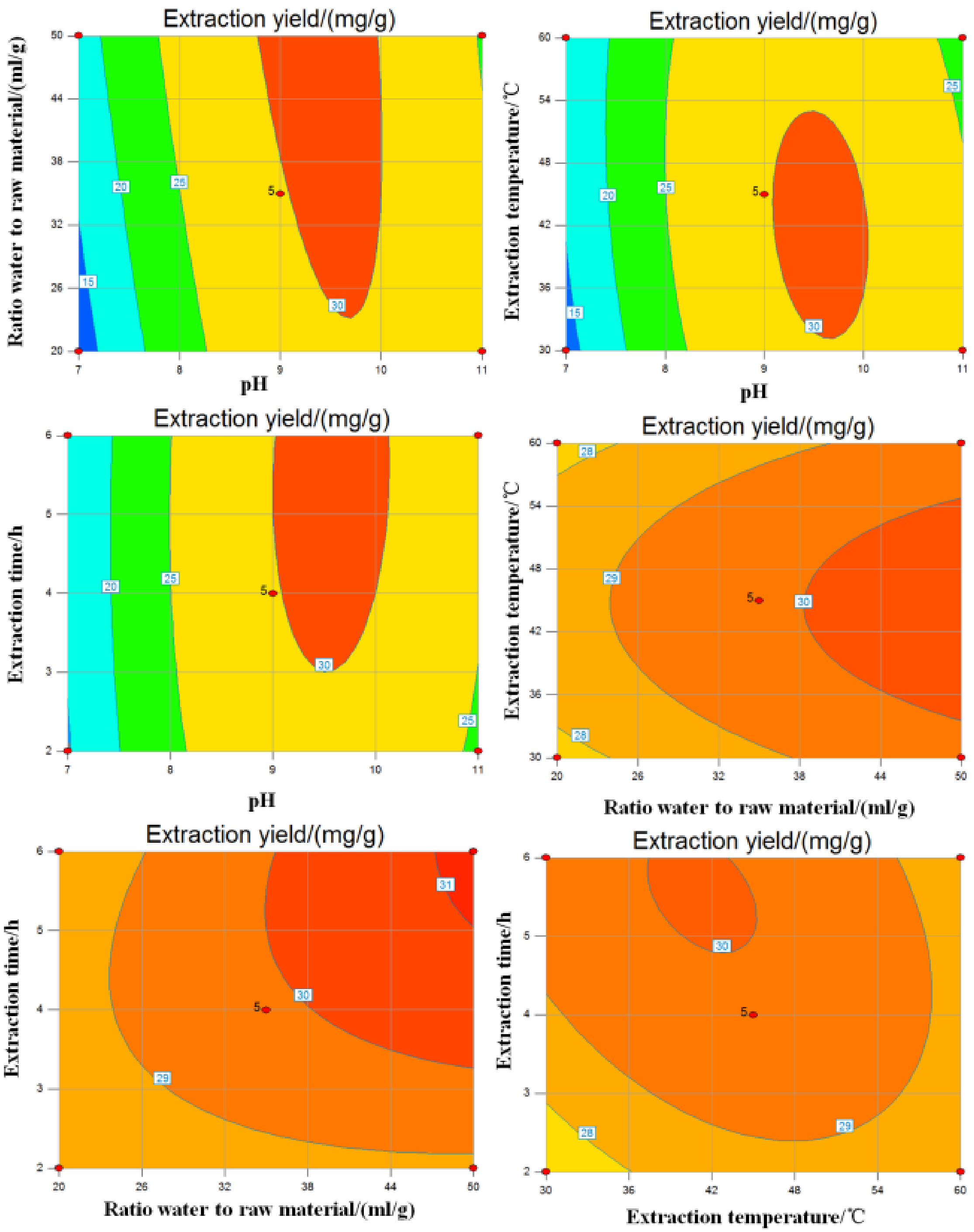


**Supp.Fig.2.** Contour plots showing the interactive effects of ratio of water to raw material and ultrasonic power (a), ratio of water to raw material and extraction temperature (b), ratio of water to raw material and extraction time (c), ultrasonic power and extraction temperature (d), ultrasonic power and extraction time (e), extraction temperature and extraction time (f) on yield of water-soluble protein

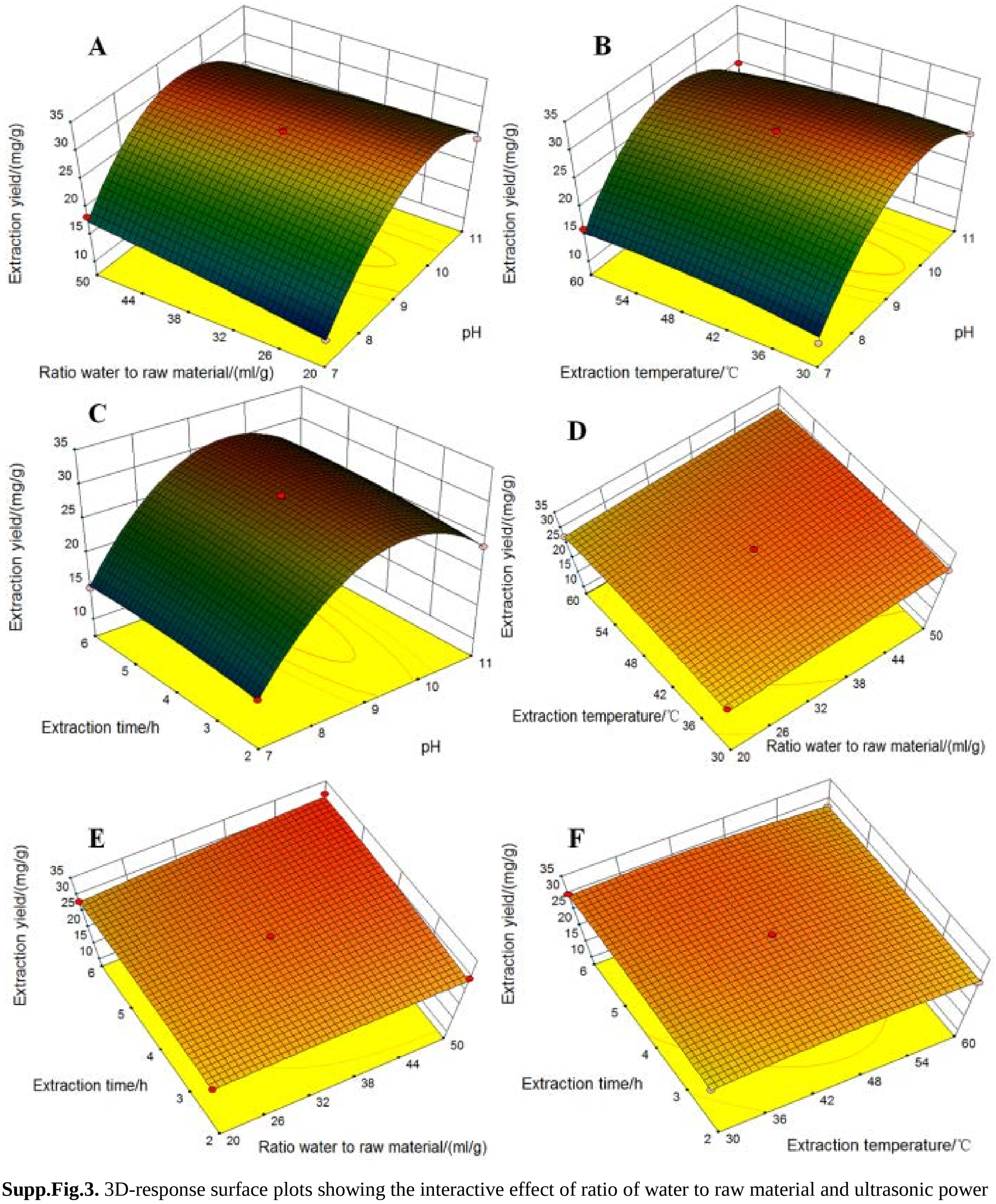


**Supp.Fig.3.** 3D-response surface plots showing the interactive effect of ratio of water to raw material and ultrasonic power (a), ratio of water to raw material and extraction temperature (b), ratio of water to raw material and extraction time (c), ultrasonic power and extraction temperature (d), ultrasonic power and extraction time (e), extraction temperature and extraction time (f) on yield of water-soluble protein
